# Anti-breast cancer sinomenine derivatives via mechanisms of apoptosis induction and metastasis reduction

**DOI:** 10.1080/14756366.2022.2096020

**Published:** 2022-07-08

**Authors:** Xiang Gao, Baojia Sun, Yonglian Hou, Lilin Liu, Jianan Sun, Fanxing Xu, Dahong Li, Huiming Hua

**Affiliations:** a Key Laboratory of Structure-Based Drug Design & Discovery, Ministry of Education, and School of Traditional Chinese Materia Medica, Shenyang Pharmaceutical University, Shenyang, China; b Yantai Valiant Pharmaceutical Co. Ltd, Shandong, China; c Wuya College of Innovation, Shenyang Pharmaceutical University, Shenyang, China

**Keywords:** Sinomenine, furoxan, breast cancer, apoptosis

## Abstract

Sinomenine, a morphinane-type isoquinoline-derived alkaloid, was first isolated from stems and roots of *Sinomenium diversifolius* (Miq.) in 1920. Later discovery by researchers confirmed various essential biological efficacy sinomenine exerted *in vitro* and *in vivo*. In this study, a series of 15 sinomenine/furoxan hybrid compounds were designed and synthesised in search of a TNBC drug candidate. Some of the target compounds exhibited strong antiproliferative activities against cancer cell lines, especially for TNBC cells, compared to positive controls. Among them, hybrid **7Cc** exerted superior cytotoxic effects on cancer cell lines with exceptionally low IC_50_ (0.82 μM) against MDA-MB-231 cells with the highest safety index score. Further studies in mechanism displayed that **7Cc** could induce an S phase cell cycle arrest, stimulate apoptosis in MDA-MB-231 cells, disrupt mitochondrial membrane potential and exert a genotoxic effect on DNA in cancer cells. In addition, **7Cc** also notably inhibited MDA-MB-231 cells in both migration, invasion and adhesion.

## 1. Introduction

Cancer resulted in nearly 10 million death in 2020 according to Globocan 2020 report^1^ with almost 20 million new cases recorded. A heavy death toll combined with a strong 5-year prevalence, especially in Asia (40.8%), Europe (26.7%) and North America (18.7%), would enable cancer one of the most dangerous health issues facing this world^1–3^. Despite global effort having been put into the development of novel therapy and application of early detection, a declining trend in the death rate for cancer overall failed to decelerate the rising incidence of breast cancer^4^. Statistics indicated that female breast cancer accounted for 11.7% of newly reported cancer cases in 2020, and overtook lung cancer by a slim margin as the most frequently diagnosed for both sex^1^. Although the estimated number of deaths in breast cancer trailed behind that of lung cancer or colorectum cancer when both genders were included, breast cancer has already become the number one cause of cancer mortality in female patients alone, projected at 684,996 deaths in 2020 around the world^1^. Hence, the alleviation of suffering sustained from breast cancer required more efficient treatment to cope with the higher incident rate, especially in resources limited low-income countries^1^^,^^4^. A key to solving these problems would be a more effective therapeutic method for treating triple-negative breast cancer (TNBC), the most metastatic and resistant type of breast cancer named for its complete deficiency in oestrogen receptor (ER), progesterone receptor (PR) plus human epidermal growth factor receptor 2 (HER2) in tumour cell surface^5–8^. These missing receptors rendered most targeted therapies effective for other breast cancer aimless while genetic instability characterised by TNBC potentially uplifted the risk of drug resistance for TNBC treatment and opened more pathways that could bypass stand-alone therapeutic methods^9–14^. The situation was further compounded by an early onset at a younger age, strong metastatic tendency, highly aggressive prognosis and often poorer clinical outcomes than other types of breast cancer^15^^,^^16^. Hence, breast cancer, TNBC especially, attracted cancer researchers around the world to better understand this disease and to find more effective treatment^16^. Many progress was already emerged such as the link between BRCA 1 and 2 mutations and TNBC, or new clinical studies included novel platinum therapy and poly-ADP ribose polymerase (PARP) inhibitors^17–20^. Yet more therapeutic options for TNBC treatment were still sorely needed, and novel medications applied in chemotherapy remained the most optimal choice against recurrence after surgery, either in stand-alone use or in therapeutic regimens^16^. It was imperative to search for novel chemical compounds with unique scaffolds both safe and open to further modification for a potential therapeutic agent in TNBC treatment. In the meantime, it would be enormously beneficial to find potential new drugs for dangerous sub-types of prevalent cancer that specifically occurred in women.

**Figure 1.**
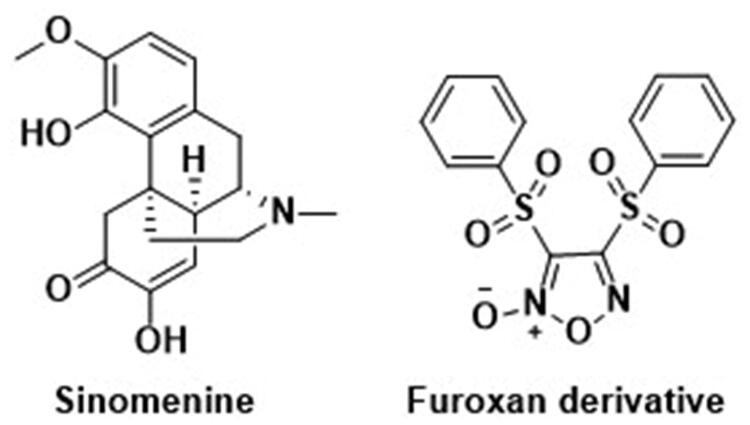
The chemical structures of reported Sinomenine and furoxan derivatives.

Almost 45% of all first-of-kind anticancer drugs approved since late 2019 owned their structures entirely or partially to natural origin^21^. Baring biological and vaccine categories, over 84% of small molecules for anti-tumour purposes exhibited a close link to the natural product and/or its derivatives, a steady climb from 77% in 2010^21^^,^^22^. Various unique structures together with a wide range of bioactivities and mechanisms unearthed from natural resources remained one of the most reliable sources that cancer drug development tapped into^23–30^. Among them, alkaloids consisted of an enormous amount of natural products with diverse scaffolds and origins^31^. Sinomenine, (7,8-didehydro-4-hydroxy-3,7-dimethoxy-17-methylmorphinan-6-one), a morphinane-type isoquinoline-derived alkaloid, was first isolated from stems and roots of *Sinomenium diversifolius* (Miq.) by Ishiwari and Nisaburo in 1920 (Figure 1)^32^. Later discovery by researchers confirmed various essential biological efficacy sinomenine exerted *in vitro* or/and *in vivo*, including analgesic activity^33–38^, anti-angiogenesis activity^39^, anticonvulsant activity^40^, anti-inflammation^41^^,^^42^, immunosuppressive activity^43^^,^^44^ and anticancer activity^45^. One particular area of study, the anticancer potential of sinomenine, gathered momentum for its ability against a panel of tumours, such as lung cancer^46^^,^^47^, liver cancer^48^, breast cancer^49^, osteosarcoma^50^, colorectal cancer^51^ and gastric cancer^52^^,^^53^. Mechanisms for the aforementioned biological effects encompassed numerous molecular targets and mechanistic pathways resulting in decreased cancer cell proliferation, induced cell cycle arrest and apoptosis. In addition, concurrent use of sinomenine with 5-Fu heightened cellular susceptibility considerably for chemotherapy, which opened the way for potential application in a combined therapeutic regimen with other anticancer drugs^49^. Despite the thorough investigation into sinomenine for its anticancer efficacy, a number of side effects still seriously hindered its clinical utilisation, including gastrointestinal disorder, insufficient biological half-life and dissatisfied physicochemical conditions^54^. However, complex ring scaffold and potential binding sites allowed space for molecular modification, at the C4 hydroxyl radical for instance, to minimise adverse effects with bespoken therapeutic ability and safety enhanced or remained intact^54^. Optimised sinomenine derivatives with strong anticancer efficacy would be apt candidates to develop potential TNBC treatment.

Nitric Oxide (NO) was a key factor in biological mediation central to many vital physiological mechanisms^55^. The aforementioned mediation abilities included homeostasis of the blood vessel, such as vasodilation, blood vessel permeability and antithrombotic effect^56^, neuromodulatory properties functioned as an on-demand neurotransmitter worked in a neuro-diffusing fashion^57^, regulation of inflammatory processes either through direct anti-oxidant ability or indirect mediation via intermediary factors released from different cell types^58^ plus notable influence over tumorigenesis and metastasis in an enzyme (mainly isoform of nitric oxide synthase) dependant way^59^. The anticancer facet of NO therapeutic potential is typically associated with a high concentration of NO in both gaseous form and metabolised form of reactive nitrogen species (RNS) binding and changing target proteins^60–64^. This shift of biological functions in these modified proteins led to a downstream cytotoxic effect against cancerous cells via apoptosis and stanched metastasis. Hence, a steady release of NO in a desirable spot of cancer for a longer period of time than the original half-life (2–30 s) was considered an apt choice of tumour treatment with clinical significance^65^^,^^66^. To achieve precise control of NO release *in situ*, NO donors were introduced with good chemical integrity and an improved method for triggering a set amount of gaseous release in enzyme dependant and/or independent way^67–69^. Many frequently used NO donors, including organic nitrate compounds, NO-metal complexes, nitrosothiols and still more to come, benefitted the research into the physiological traits and therapeutic potentials of NO in both cellulars and *in vivo* carcinogenic models profoundly^70^^,^^71^. Among them, furoxan exhibited conspicuous anticancer ability in subsequent studies under a GST-positive condition against certain tumour cell lines since its first report in 1981 (Figure 1)^72^. The diminished effect of furoxan in the neoplastic environment with additional NO scavengers (such as hemoglobin) affirmed the link between the NO level maintained by furoxan and the anticancer effect displayed concomitantly^73^. In addition, different from other NO donating molecules, pharmacological studies revealed furoxan did not prone to acquire multi-drug resistance in the course of cancer therapy, and several furoxan/natural product hybrids seamlessly grafted potent anticancer effect and selectivity from each other into one drug candidate. Therefore, furoxan as a pharmacophore for drug development was a sound strategy in search of novel treatment against breast cancer, especially drug-resistant TNBC.

In a nutshell, this proposed union between biologically versatile natural product building block and therapeutically potent NO donor pharmacophore ushered in a promising strategy for finding breast cancer drug candidates which could be particularly effective against TNBC. Herein, a series of novel sinomenine/furoxan hybrids were designed and synthesised, their anticancer abilities were extensively examined through various experiments to establish potential anticancer efficacy.

## 2. Experimental

### 2.1. Chemistry

Commercial suppliers provided all synthetic components and reagents directly, these materials were utilised in the synthetic process with no need for extra purification. A portion of anhydrous reagents was self-prepared for laboratory-wide use, synthesis in this article included. A Bruker ARX-400 NMR spectrometer (Bruker, Karlsruhe, Germany) recorded the measurement of ^1^H and ^13 ^C NMR spectra for all compounds. The internal standard was selected as tetramethylsilane (TMS) and *δ* was chosen to represent chemical shifts in NMR reports. An Agilent QTOF6520 high-resolution mass spectrometer (Agilent Technologies, Palo Alto, CA) recorded the measurement of high-resolution mass spectra (HR-MS) data from all tested compounds.

#### 2.1.1. General procedures for the synthesis of compounds 7A–F(a–c)

Adding 10% NaOH to the liquid of thiophenol for a stir of 15 min, followed by chloroacetic acid at 140 °C for 4 h. Then, acidify the reaction till pH = 2, and place the reaction in to sub-0 °C environment for crystallisation. And compound **2** was acquired through filtering. 2-Phenylsulfanylacetic acid was resolved in acetic acid and then 30% H_2_O_2_ was added dropwise for a 3 h stir. The fuming nitric acid was added in drop after a 0 °C cooling was observed in this reaction, and then increasing the reaction temperature to 100 °C for 6 h stirring. Cool the reaction till crystallisation occurred and 1,2,5-oxadiazole **4** was acquired via filtering.

Compound **4** was dissolved in tetrahydrofuran at 0 °C and followed by corresponding diol agents. Then, the reaction was cooled down to −10 °C before 30% NaOH was added. Upon completion, the THF in this mixture was replaced by dichloromethane for extraction from water. The DCM was combined, washed with brine, and dried in anhydrous Na_2_SO_4_, then the solvent was removed *in vacuo* to attain a crude product. The intermediary has then undergone silica gel column chromatography for further purification (DCM/MeOH system) and pure product **5A**–**F** were acquired.

Product **5A**–**F** were mixed with the corresponding anhydride in DCM, then followed by the addition of triethylamine and DMAP for 2 h. Upon completion, water was added for extraction. The DCM was combined, washed with brine, and dried in anhydrous Na_2_SO_4_, then the solvent was removed *in vacuo* to attain a crude product. The target molecule was undergone silica gel column chromatography for further purification (DCM/MeOH system) and pure product **6A**–**F**(**a**–**c**) was acquired.

Sinomenine was placed in DCM then followed by a corresponding intermediary with EDCI and DMAP for a 4 h reaction. Upon completion, water was added for extraction. The DCM was combined, washed with brine, and dried in anhydrous Na_2_SO_4_, then the solvent was removed *in vacuo* to attain a crude product. The target molecule was undergone silica gel column chromatography for further purification (DCM/MeOH system) and pure product **7A**–**F**(**a**–**c**) was acquired.

#### 2.1.2. 4–(2-((4-(((4bs,9R)-3,7-Dimethoxy-11-methyl-6-oxo-6,8a,9,10-tetrahydro-5H-9,4b-(epiminoethano)phenanthren-4-yl)oxy)-4-oxobutanoyl)oxy)ethoxy)-3-(phenylsulfonyl)-1,2,5-oxadiazole 2-oxide (7Aa)

Light yellow solid, 63.4% yield. ^1^H NMR (CDCl_3_, 400 MHz) *δ*: 8.06 (m, 2H, H-1′,5′), 7.73 (m, 1H, H-3′), 7.61 (m, 2H, H-2′,4′), 6.91 (d, *J* = 8.4 Hz, 1H, H-1), 6.74 (d, *J* = 8.5 Hz, 1H, H-2), 5.46 (d, *J* = 2.1 Hz, 1H, H-8), 4.62 (m, 2H, -CH_2_-), 4.54 (m, 2H, -CH_2_-), 3.72 (s, 3H, -OCH_3_-), 3.46 (s, 3H, -OCH_3_-), 3.19 (t, *J* = 4.3 Hz, 1H, H-9), 3.01 (m, 2H, -CH_2_-), 2.74 (m, 3H, H-14, -CH_2_-), 2.50 (m, 2H, -CH_2_-), 2.50 (m, 2H, -CH_2_-), 2.43 (s, 3H, -CH_3_-), 1.89 (m, 2H, -CH_2_-), 1.61 (m, 2H, -CH_2_-); ^13 ^C NMR (CDCl_3_, 100 MHz) *δ*: 192.3, 172.3, 170.4, 158.6, 152.4, 149.6, 139.4, 137.9, 135.6, 129.6, 128.5, 125.4, 114.8, 110.8, 110.3, 68.8, 61.4, 56.3, 55.9, 50.0, 46.6, 45.8, 42.6, 40.6, 29.6, 29.2, 28.8, 24.2; HRMS (ESI) *m/z* calcd for C_33_H_35_N_3_O_12_S [M + H]^+^ 698.2020, found 698.2014.

#### 2.1.3. 4–(2-((5-(((4bs,9R)-3,7-Dimethoxy-11-methyl-6-oxo-6,8a,9,10-tetrahydro-5H-9,4b-(epiminoethano)phenanthren-4-yl)oxy)-5-oxopentanoyl)oxy)ethoxy)-3-(phenylsulfonyl)-1,2,5-oxadiazole 2-oxide (7Ab)

Light yellow solid, 54.7% yield. ^1^H NMR (CDCl_3_, 400 MHz) *δ*: 8.06 (m, 2H, H-1′,5′), 7.72 (m, 1H, H-3′), 7.60 (m, 2H, H-2′,4′), 6.90 (d, *J* = 8.4 Hz, 1H, H-1), 6.74 (d, *J* = 8.5 Hz, 1H, H-2), 5.46 (d, *J* = 2.1 Hz, 1H, H-8), 4.64 (dd, *J* = 5.3, 3.8 Hz, 2H, -CH_2_-), 4.54 (m, 2H, -CH_2_-), 3.71 (s, 3H, -OCH_3_-), 3.46 (s, 3H, OCH_3_-), 3.18 (t, *J* = 4.3 Hz, 1H, H-9), 3.04 (m, 4H, -CH_2_-), 2.74 (m, 3H, H-14, -CH_2_-), 2.59 (m, 2H, -CH_2_-), 2.43 (s, 3H, -CH_3_-), 2.13 (m, 4H, -CH_2_-), 1.61 (m, 2H, -CH_2_-); ^13 ^C NMR (CDCl_3_, 100 MHz) *δ*: 192.6, 173.0, 171.7, 159.0, 152.8, 150.0, 139.7, 138.3, 136.0, 130.0, 129.8, 128.7, 125.8, 115.2, 111.2, 110.8, 69.3, 61.5, 56.7, 56.2, 55.2, 54.8, 50.5, 46.9, 46.1, 43.0, 40.9, 37.4, 33.7, 33.2, 24.6, 20.4; HRMS (ESI) *m/z* calcd for C_34_H_37_N_3_O_12_S [M + H]^+^ 712.2176, found 712.2171.

#### 2.1.4. 4–(2-((2-((((4bs,9R)-3,7-Dimethoxy-11-methyl-6-oxo-6,8a,9,10-tetrahydro-5H-9,4b-(epiminoethano)phenanthren-4-yl)oxy)carbonyl)benzoyl)oxy)ethoxy)-3-(phenylsulfonyl)-1,2,5-oxadiazole 2-oxide (7Ac)

Light yellow solid, 57.8% yield. ^1^H NMR (CDCl_3_, 400 MHz) *δ*: 8.41 (d, *J* = 7.3 Hz, 1H, H-4′), 8.01 (m, 2H, H-2′',4′'), 7.70 (m, 3H, H-1′,1′',5′'), 7.63 (m, 1H, H-3′'), 7.42 (m, 2H, H-2′,3′), 6.95 (d, *J* = 8.4 Hz, 1H, H-1), 6.78 (d, *J* = 8.4 Hz, 1H, H-2), 5.47 (d, *J* = 2.1 Hz, 1H, H-8), 4.68 (m, 4H, -CH_2_-), 3.67 (s, 3H, -OCH_3_-), 3.48 (s, 3H, -OCH_3_-), 3.21 (t, *J* = 4.4 Hz, 1H, H-9), 3.05 (m, 2H, CH_2_), 2.77 (m, 1H, H-14), 2.51 (m, 2H, -CH_2_-), 2.43 (m, 3H, -CH_3_-), 2.18 (m, 2H, -CH_2_-), 1.87 (m, 2H, -CH_2_-);^13^C NMR (CDCl_3_, 100 MHz) *δ*: 192.7, 168.2, 164.0, 158.8, 152.8, 149.8, 139.6, 138.2, 135.7, 134.4, 132.8, 131.4, 130.5, 130.0, 129.7, 129.1, 128.7, 128.5, 125.9, 114.9, 111.2, 110.5, 68.9, 62.4, 56.6, 56.1, 55.0, 50.3, 46.8, 46.0, 42.8, 40.9, 37.0, 29.8, 24.4; HRMS (ESI) *m/z* calcd for C_37_H_35_N_3_O_12_S [M + H]^+^ 746.2020, found 746.2014.

#### 2.1.5. (6-Methyl-4-oxo-4H-chromen-3-yl)methyl 4–(3-((4-(((4bS,9R)-3,7-dimethoxy-11-methyl-6-oxo-6,8a,9,10-tetrahydro-5H-9,4b-(epiminoethano)phenanthren-4-yl)oxy)-4-oxobutanoyl)oxy)propoxy)-3-(phenylsulfonyl)-1,2,5-oxadiazole 2-oxide (7Ba)

Light yellow solid, 66.4% yield. ^1^H NMR (CDCl_3_, 400 MHz) *δ*: 8.05 (m, 2H, H-1′,5′), 7.73 (m, 1H, H-3′), 7.61 (m, 2H, H-2′,4′), 6.89 (d, *J* = 8.4 Hz, 1H, H-1), 6.73 (d, *J* = 8.5 Hz, 1H, H-2), 5.46 (d, *J* = 2.1 Hz, 1H, H-8), 4.50 (m, 2H, -CH_2_-), 4.31 (m, 2H, -CH_2_-), 3.71 (s, 3H, -OCH_3_-), 3.47 (s, 3H, -OCH_3_-), 3.17 (t, *J* = 4.3 Hz, 1H, H-9), 3.01(m, 4H, -CH_2_-), 2.74 (m, 3H, H-14, -CH_2_-), 2.49 (m, 2H, -CH_2_-), 2.42 (s, 3H, -CH_3_-), 2.10 (m, 2H, -CH_2_-), 1.89 (m, 2H, -CH_2_-), 1.61 (m, 2H, -CH_2_-); ^13 ^C NMR (CDCl_3_, 100 MHz) *δ*: 192.5, 172.2, 170.5, 158.9, 152.6 149.8, 139.6, 138.2, 135.8, 129.8, 128.7, 125.6, 115.1, 111.0, 110.6, 68.1, 60.7, 56.5, 56.1, 55.0, 50.3, 46.8, 46.1, 42.8, 40.8, 37.2, 29.5, 29.1, 28.1, 24.4; HRMS (ESI) *m/z* calcd for C_34_H_37_N_3_O_12_S [M + H]^+^ 712.2176, found 712.2171.

#### 2.1.6. 4–(3-((5-(((4bs,9R)-3,7-Dimethoxy-11-methyl-6-oxo-6,8a,9,10-tetrahydro-5H-9,4b-(epiminoethano)phenanthren-4-yl)oxy)-5-oxopentanoyl)oxy)propoxy)-3-(phenylsulfonyl)-1,2,5-oxadiazole 2-oxide (7Bb)

Light yellow solid, 53.8% yield. ^1^H NMR (CDCl_3_, 400 MHz) *δ*: 8.04 (m, 2H, H-1′,5′), 7.73 (m, 1H, H-3′), 7.61 (m, 2H, H-2′,4′), 6.89 (d, *J* = 8.4 Hz, 1H, H-1), 6.73 (d, *J* = 8.5 Hz, 1H, H-2), 5.46 (d, *J* = 2.1 Hz, 1H, H-8), 4.50 (dd, *J* = 5.3, 3.8 Hz, 2H, -CH_2_-), 4.28 (m, 2H, -CH_2_-), 3.70 (s, 3H, -OCH_3_-), 3.45 (s, 3H, -OCH_3_-), 3.16 (t, *J* = 4.3 Hz, 1H, H-9), 3.03 (m, 2H, -CH_2_-), 2.70 (m, 3H, H-14, -CH_2_-), 2.50 (m, 4H, -CH_2_-), 2.43 (s, 3H, -CH_3_-), 2.13 (m, 6H, -CH_2_-), 1.61 (m, 2H, -CH_2_-);^13^C NMR (CDCl_3_, 100 MHz) *δ*: 192.3, 172.9, 170.9, 158.9, 152.6, 149.7, 139.5, 138.1, 135.8, 129.8, 128.6, 125.5, 115.1, 110.9, 110.5, 68.1, 60.3, 56.4, 56.0, 54.9, 50.3, 46.7, 45.9, 42.7, 40.7, 37.2, 33.5, 33.1, 28.1, 24.3, 20.2; HRMS (ESI) *m/z* calcd for C_35_H_39_N_3_O_12_S [M + H]^+^ 726.2333, found 726.2327.

#### 2.1.7. 4–(3-((2-((((4bs,9R)-3,7-Dimethoxy-11-methyl-6-oxo-6,8a,9,10-tetrahydro-5H-9,4b-(epiminoethano)phenanthren-4-yl)oxy)carbonyl)benzoyl)oxy)propoxy)-3-(phenylsulfonyl)-1,2,5-oxadiazole 2-oxide (7Bc)

Light yellow solid, 47.9% yield. ^1^H NMR (CDCl_3_, 400 MHz) *δ*: 8.43 (d, *J* = 7.3 Hz, 1H, H-4′), 8.03 (m, 2H, H-2′',4′'), 7.67 (m, 3H, H-1′,1′',5′'), 7.63 (m, 1H, H-3′'), 7.42 (m, 2H, H-2′,3′), 6.94 (d, *J* = 8.4 Hz, 1H, H-1), 6.77 (d, *J* = 8.4 Hz, 1H, H-2), 5.48 (d, *J* = 2.1 Hz, 1H, H-8), 4.45 (m, 4H, -CH_2_-), 3.69 (s, 3H, -OCH_3_-), 3.48 (s, 3H, -OCH_3_-), 3.18 (t, *J* = 4.4 Hz, 1H, H-9), 3.05 (m, 2H, -CH_2-_), 2.74 (m, 1H, H-14), 2.51 (m, 2H, -CH_2_-), 2.43 (m, 3H, -CH_3_-), 2.18 (m, 2H, -CH_2_-), 1.87 (m, 2H, -CH_2_-), 1.73 (m, 2H, -CH_2_-); ^13 ^C NMR (CDCl_3_, 100 MHz) *δ*: 192.8, 168.7, 164.1, 158.9, 152.7, 149.7, 139.5, 138.2, 135.7, 135.1, 132.8, 131.4, 131.1, 130.5, 130.1, 129.8, 128.6, 128.3, 125.8, 115.1, 111.0, 110.5, 68.1, 61.5, 56.5, 56.1, 55.0, 50.5, 46.7, 46.2, 42.8, 41.0, 37.1, 29.8, 28.0, 24.3; HRMS (ESI) *m/z* calcd for C_38_H_37_N_3_O_12_S [M + H]^+^ 760.2176, found 760.2171.

#### 2.1.8. 4–(4-((4-(((4bs,9R)-3,7-Dimethoxy-11-methyl-6-oxo-6,8a,9,10-tetrahydro-5H-9,4b-(epiminoethano)phenanthren-4-yl)oxy)-4-oxobutanoyl)oxy)butoxy)-3-(phenylsulfonyl)-1,2,5-oxadiazole 2-oxide (7Ca)

Light yellow solid, 51.3% yield. ^1^H NMR (CDCl_3_, 400 MHz) *δ*: 8.08 (m, 2H, H-1′, 5′), 7.72 (m, 1H, H-3′), 7.63 (m, 2H, H-2′,4′), 6.87 (d, *J* = 8.4 Hz, 1H, H-1), 6.73 (d, *J* = 8.5 Hz, 1H, H-2), 5.44 (d, *J* = 2.1 Hz, 1H, H-8), 4.42 (m, 2H, -CH_2_-), 4.13 (m, 2H,-CH_2_-), 3.71 (s, 3H, -OCH_3_-), 3.47 (s, 3H, -OCH_3_-), 3.17 (t, *J* = 4.3 Hz, 1H, H-9), 2.97 (m, 4H, -CH_2_-), 2.76 (m, 3H, H-14, -CH_2_-), 2.51 (m, 2H, -CH_2_-), 2.42 (s, 3H, -CH_3_-), 1.95 (m, 4H, -CH_2_-), 1.82 (m, 2H, -CH_2_-), 1.61 (m, 2H, -CH_2_-); ^13 ^C NMR (CDCl_3_, 100 MHz) *δ*: 192.5, 172.1, 170.6, 158.9, 152.5, 149.7, 139.5, 138.1, 135.7, 130.1, 129.9, 129.7, 128.5, 125.5, 115.0, 110.9, 110.5, 71.0, 63.9, 56.4, 56.0, 54.8, 50.1, 46.7, 46.0, 42.7, 40.7, 37.0, 29.4, 29.0, 25.6, 25.4, 24.4; HRMS (ESI) *m/z* calcd for C_35_H_39_N_3_O_12_S [M + H]^+^ 726.2333, found 726.2327.

#### 2.1.9. 4–(4-((5-(((4bs,9R)-3,7-Dimethoxy-11-methyl-6-oxo-6,8a,9,10-tetrahydro-5H-9,4b-(epiminoethano)phenanthren-4-yl)oxy)-5-oxopentanoyl)oxy)butoxy)-3-(phenylsulfonyl)-1,2,5-oxadiazole 2-oxide (7Cb)

Light yellow solid, 60.6% yield. ^1^H NMR (CDCl_3_, 400 MHz) *δ*: 8.05 (m, 2H, H-1′,5′), 7.74 (m, 1H, H-3′), 7.61 (m, 2H, H-2′,4′), 6.89 (d, *J* = 8.4 Hz, 1H, H-1), 6.74 (d, *J* = 8.5 Hz, 1H, H-2), 5.46 (d, *J* = 2.1 Hz, 1H, H-8), 4.42 (m, 2H, -CH_2_-), 4.19 (m, 2H,-CH_2_-), 3.71 (s, 3H, -OCH_3_-), 3.47 (s, 3H, -OCH_3_-), 3.18 (t, *J* = 4.3 Hz, 1H, H-9), 3.03 (m, 2H, -CH_2_-), 2.74 (m, 3H, H-14, -CH_2_-), 2.51 (m, 4H, -CH_2_-), 2.43 (s, 3H, -CH_3_-), 2.11 (m, 4H, -CH_2_-), 1.84 (m, 4H, -CH_2_-), 1.61 (m, 2H, -CH_2_-); ^13 ^C NMR (CDCl_3_, 100 MHz) *δ*: 192.3, 172.2, 170.6, 158.9, 152.6, 149.7, 139.5, 138.4, 135.6, 130.3, 129.9, 129.7, 128.8, 125.5, 115.0, 110.9, 110.5, 71.0, 63.9, 56.5, 56.0, 54.8, 50.3, 46.9, 46.0, 42.6, 40.7, 37.0, 29.4, 29.0, 25.6, 25.4, 24.4; HRMS (ESI) *m/z* calcd for C_36_H_41_N_3_O_12_S [M + H]^+^ 740.2489, found 740.2484.

#### 2.1.10. 4–(4-((2-((((4bs,9R)-3,7-Dimethoxy-11-methyl-6-oxo-6,8a,9,10-tetrahydro-5H-9,4b-(epiminoethano)phenanthren-4-yl)oxy)carbonyl)benzoyl)oxy)butoxy)-3-(phenylsulfonyl)-1,2,5-oxadiazole 2-oxide (7Cc)

Light yellow solid, 60.5% yield. ^1^H NMR (CDCl_3_, 400 MHz) *δ*: 8.38 (d, *J* = 7.3 Hz, 1H, H-4′), 8.02 (m, 2H, H-2′',4′'), 7.72 (m, 3H, H-1′, 1′',5′'), 7.63 (m, 1H, H-3′'), 7.42 (m, 2H, H-2′,3′), 6.93 (d, *J* = 8.4 Hz, 1H, H-1), 6.78 (d, *J* = 8.4 Hz, 1H, H-2), 5.48 (d, *J* = 2.1 Hz, 1H, H-8), 4.40 (m, 4H, -CH_2_-), 3.71 (s, 3H, -OCH_3_-), 3.48 (s, 3H, -OCH_3_-), 3.22 (t, *J* = 4.4 Hz, 1H, H-9), 3.06 (m, 2H, CH_2_), 2.78 (m, 1H, H-14), 2.48 (m, 2H, -CH_2_-), 2.46 (m, 3H, -CH_3_-), 1.96 (m, 4H, -CH_2_-), 1.88 (m, 4H, -CH_2_-); ^13 ^C NMR (CDCl_3_, 100 MHz) *δ*: 192.8, 168.7, 164.1, 158.9, 152.7, 149.7, 139.5, 138.2, 135.7, 135.1, 132.8, 131.4, 131.1, 130.5, 130.1, 129.8, 128.6, 128.3, 125.8, 115.1, 111.0, 110.5, 71.6, 65.6, 56.6, 56.1, 55.0, 50.3, 46.9, 46.1, 42.8, 40.9, 37.0, 25.2, 24.8, 24.4; HRMS (ESI) *m/z* calcd for C_39_H_39_N_3_O_12_S [M + H]^+^ 774.2333, found 774.2327.

#### 2.1.11. 4-((5-((4-(((4bs,9R)-3,7-Dimethoxy-11-methyl-6-oxo-6,8a,9,10-tetrahydro-5H-9,4b-(epiminoethano)phenanthren-4-yl)oxy)-4-oxobutanoyl)oxy)pentyl)oxy)-3-(phenylsulfonyl)-1,2,5-oxadiazole 2-oxide (7 Da)

Light yellow solid, 70.7% yield. ^1^H NMR (CDCl_3_, 400 MHz) *δ*: 8.05 (m, 2H, H-1′,5′), 7.73 (m, 1H, H-3′), 7.62 (m, 2H, H-2′,4′), 6.85 (d, *J* = 8.4 Hz, 1H, H-1), 6.72 (d, *J* = 8.5 Hz, 1H, H-2), 5.43 (d, *J* = 2.1 Hz, 1H, H-8), 4.44 (m, 2H, -CH_2_-), 4.16 (m, 2H,-CH_2_-), 3.71 (s, 3H, -OCH_3_-), 3.47 (s, 3H, -OCH_3_-), 3.22 (t, *J* = 4.3 Hz, 1H, H-9), 2.96 (m, 4H, -CH_2_-), 2.79 (m, 3H, H-14, -CH_2_-), 2.54 (m, 2H, -CH_2_-), 2.45 (s, 3H, -CH_3_-), 2.12 (m, 2H, -CH_2_-), 1.82 (m, 2H, -CH_2_-), 1.61 (m, 2H, -CH_2_-), 1.54 (m, 2H, -CH_2_-); ^13 ^C NMR (CDCl_3_, 100 MHz) *δ*: 192.5, 172.1, 170.6, 158.9, 152.5, 149.7, 139.5, 138.1, 135.7, 130.1, 129.9, 129.7, 128.5, 125.5, 115.0, 110.9, 110.5, 71.0, 63.9, 56.4, 56.0, 54.8, 50.1, 46.7, 46.0, 42.7, 40.7, 37.0, 29.4, 29.0, 28.1, 25.6, 25.4, 24.4; HRMS (ESI) *m/z* calcd for C_36_H_41_N_3_O_12_S [M + H]^+^ 740.2489, found 740.2484.

#### 2.1.12. 4-((5-((5-(((4bs,9R)-3,7-Dimethoxy-11-methyl-6-oxo-6,8a,9,10-tetrahydro-5H-9,4b-(epiminoethano)phenanthren-4-yl)oxy)-5-oxopentanoyl)oxy)pentyl)oxy)-3-(phenylsulfonyl)-1,2,5-oxadiazole 2-oxide (7Db)

Light yellow solid, 63.1% yield. ^1^H NMR (CDCl_3_, 400 MHz) *δ*: 8.04 (m, 2H, H-1′,5′), 7.75 (m, 1H, H-3′), 7.62 (m, 2H, H-2′,4′), 6.90 (d, *J* = 8.4 Hz, 1H, H-1), 6.75 (d, *J* = 8.5 Hz, 1H, H-2), 5.46 (d, *J* = 2.1 Hz, 1H, H-8), 4.41 (m, 2H, -CH_2_-), 4.15 (m, 2H,-CH_2_-), 3.72 (s, 3H, -OCH_3_-), 3.47 (s, 3H, -OCH_3_-), 3.20 (t, *J* = 4.3 Hz, 1H, H-9), 3.03 (m, 2H, -CH_2_-), 2.74 (m, 3H, H-14, -CH_2_-), 2.51 (m, 4H, -CH_2_-), 2.45 (s, 3H, -CH_3_-), 2.11 (m, 4H, -CH_2_-), 1.90 (m, 4H, -CH_2_-), 1.75 (m, 2H, -CH_2_-), 1.56 (m, 2H, -CH_2_-); ^13 ^C NMR (CDCl_3_, 100 MHz) *δ*: 192.5, 172.1, 170.6, 158.9, 152.5, 149.7, 139.5, 138.1, 135.7, 130.1, 129.9, 129.7, 128.5, 125.5, 115.0, 110.9, 110.5, 71.0, 63.9, 56.4, 56.0, 54.8, 50.1, 46.7, 46.0, 42.7, 40.7, 37.0, 29.4, 29.0, 28.1, 25.6, 25.4, 24.3, 22.3; HRMS (ESI) *m/z* calcd for C_37_H_43_N_3_O_12_S [M + H]^+^ 754.2646, found 754.2640.

#### 2.1.13. 4-((5-((2-((((4bs,9R)-3,7-Dimethoxy-11-methyl-6-oxo-6,8a,9,10-tetrahydro-5H-9,4b-(epiminoethano)phenanthren-4-yl)oxy)carbonyl)benzoyl)oxy)pentyl)oxy)-3-(phenylsulfonyl)-1,2,5-oxadiazole 2-oxide (7Dc)

Light yellow solid, 59.5% yield. ^1^H NMR (CDCl_3_, 400 MHz) *δ*: 8.36 (d, *J* = 7.3 Hz, 1H, H-4′), 8.01 (m, 2H, H-2′',4′'), 7.71 (m, 3H, H-1′,1′',5′'), 7.63 (m, 1H, H-3′'), 7.58 (m, 2H, H-2′,3′), 6.92 (d, *J* = 8.4 Hz, 1H, H-1), 6.78 (d, *J* = 8.4 Hz, 1H, H-2), 5.48 (d, *J* = 2.1 Hz, 1H, H-8), 4.37 (m, 4H, -CH_2_-), 3.71 (s, 3H, -OCH_3_-), 3.49 (s, 3H, -OCH_3_-), 3.21 (t, *J* = 4.4 Hz, 1H, H-9), 3.06 (m, 2H, CH_2_), 2.79 (m, 1H, H-14), 2.48 (m, 2H, -CH_2_-), 2.45 (m, 3H, -CH_3_-), 2.20 (m, 2H, -CH_2_-), 1.86 (m, 6H, -CH_2_-), 1.54 (m, 2H, -CH_2_-); ^13 ^C NMR (CDCl_3_, 100 MHz) *δ*: 192.8, 168.7, 164.1, 158.9, 152.7, 149.7, 139.5, 138.2, 135.7, 135.1, 132.8, 131.4, 131.1, 130.5, 130.1, 129.8, 128.6, 128.3, 125.8, 115.1, 111.0, 110.5, 71.6, 65.6, 56.6, 56.1, 55.0, 50.3, 46.9, 46.1, 42.8, 40.9, 37.0, 29.7, 28.0, 24.3, 22.1; HRMS (ESI) *m/z* calcd for C_40_H_41_N_3_O_12_S [M + H]^+^ 788.2489, found 788.2484.

#### 2.1.14. 4-((6-((4-(((4bs,9R)-3,7-Dimethoxy-11-methyl-6-oxo-6,8a,9,10-tetrahydro-5H-9,4b-(epiminoethano)phenanthren-4-yl)oxy)-4-oxobutanoyl)oxy)hexyl)oxy)-3-(phenylsulfonyl)-1,2,5-oxadiazole 2-oxide (7Ea)

Light yellow solid, 61.1% yield. ^1^H NMR (CDCl_3_, 400 MHz) *δ*: 8.05 (m, 2H, H-1′,5′), 7.74 (m, 1H, H-3′), 7.61 (m, 2H, H-2′,4′), 6.89 (d, *J* = 8.4 Hz, 1H, H-1), 6.73 (d, *J* = 8.5 Hz, 1H, H-2), 5.46 (d, *J* = 2.1 Hz, 1H, H-8), 4.40 (m, 2H, -CH_2_-), 4.12 (m, 2H, -CH_2_-), 3.70 (s, 3H, -OCH_3_-), 3.47 (s, 3H, -OCH_3_-), 3.18 (t, *J* = 4.3 Hz, 1H, H-9), 3.00 (m, 4H, -CH_2_-), 2.76 (m, 3H, H-14, -CH_2_-), 2.50 (m, 2H, -CH_2_-), 2.42 (s, 3H, -CH_3_-), 2.15 (m, 4H, -CH_2_-), 1.89 (m, 4H, -CH_2_-), 1.61 (m, 4H, -CH_2_-); ^13 ^C NMR (CDCl_3_, 100 MHz) *δ*: 192.5, 172.3, 170.3, 159.1, 152.6, 149.9, 139.7, 138.3, 135.7, 129.8, 128.6, 125.6, 115.0, 111.0, 110.6, 71.6, 64.7, 56.6, 56.1, 55.0, 50.2, 46.8, 46.0, 42.8, 40.8, 37.1, 29.6, 29.2, 28.6, 28.4, 25.6, 25.4, 24.41; HRMS (ESI) *m/z* calcd for C_37_H_43_N_3_O_12_S [M + H]^+^ 754.2646, found 754.2640.

#### 2.1.15. 4-((6-((5-(((4bs,9R)-3,7-Dimethoxy-11-methyl-6-oxo-6,8a,9,10-tetrahydro-5H-9,4b-(epiminoethano)phenanthren-4-yl)oxy)-5-oxopentanoyl)oxy)hexyl)oxy)-3-(phenylsulfonyl)-1,2,5-oxadiazole 2-oxide (7Eb)

Light yellow solid, 70.6% yield. ^1^H NMR (CDCl_3_, 400 MHz) *δ*: 8.05 (m, 2H, H-1′,5′), 7.75 (m, 1H, H-3′), 7.60 (m, 2H, H-2′,4′), 6.89 (d, *J* = 8.4 Hz, 1H, H-1), 6.74 (d, *J* = 8.5 Hz, 1H, H-2), 5.46 (d, *J* = 2.1 Hz, 1H, H-8), 4.40 (m, 2H, -CH_2_-), 4.12 (m, 2H, -CH_2_-), 3.71 (s, 3H, -OCH_3_-), 3.46 (s, 3H, -OCH_3_-), 3.19 (t, *J* = 4.3 Hz, 1H, H-9), 3.02 (m, 2H, -CH_2_-), 2.69 (m, 3H, H-14, -CH_2_-), 2.48 (m, 4H, -CH_2_-), 2.43 (s, 3H, -CH_3_-), 2.16 (m, 2H, -CH_2_-), 2.15 (m, 4H, -CH_2_-), 1.89 (m, 4H, -CH_2_-), 1.61 (m, 4H, -CH_2_-); ^13 ^C NMR (CDCl_3_, 100 MHz) *δ*: 192.3, 173.2, 170.3,159.2, 152.6, 149.9, 139.6, 138.3, 135.7, 129.8, 128.6, 125.5, 115.0, 111.0, 110.6, 71.6, 64.4, 56.6, 56.0, 55.0, 50.3, 46.8, 46.0, 42.8, 40.7, 37.2, 33.6, 33.3, 28.7, 28.4, 25.6, 25.4, 24.4, 20.3; HRMS (ESI) *m/z* calcd for C_38_H_45_N_3_O_12_S [M + H]^+^ 768.2802, found 768.2797.

#### 2.1.16. 4-((6-((2-((((4bs,9R)-3,7-Dimethoxy-11-methyl-6-oxo-6,8a,9,10-tetrahydro-5H-9,4b-(epiminoethano)phenanthren-4-yl)oxy)carbonyl)benzoyl)oxy)hexyl)oxy)-3-(phenylsulfonyl)-1,2,5-oxadiazole 2-oxide (7Ec)

Light yellow solid, 71.2% yield. ^1^H NMR (CDCl_3_, 400 MHz) *δ*: 8.36 (d, *J* = 7.3 Hz, 1H, H-4′), 8.04 (m, 2H, H-2′',4′'), 7.72 (m, 3H, H-1′,1′',5′'), 7.63 (m, 1H, H-3′'), 7.42 (m, 2H, H-2′,3′), 6.93 (d, *J* = 8.4 Hz, 1H, H-1), 6.77 (d, *J* = 8.4 Hz, 1H, H-2), 5.48 (d, *J* = 2.1 Hz, 1H, H-8), 4.32 (m, 4H, -CH_2_-), 3.70 (s, 3H, -OCH_3_-), 3.48 (s, 3H, -OCH_3_-), 3.20 (t, *J* = 4.4 Hz, 1H, H-9), 3.04 (m, 2H, CH_2_), 2.75 (m, 1H, H-14), 2.53 (m, 2H, -CH_2_-), 2.43 (m, 3H, -CH_3_-), 2.18 (m, 2H, -CH_2_-), 1.83 (m, 6H, -CH_2_-), 1.45 (m, 4H, -CH_2_-); ^13 ^C NMR (CDCl_3_, 100 MHz) *δ*: 192.8, 168.7, 164.1, 158.9, 152.7, 149.7, 139.5, 138.2, 135.7, 135.1, 132.8, 131.4, 131.1, 130.5, 130.1, 129.8, 128.6, 128.3, 125.8, 115.1, 111.0, 110.5, 71.6, 65.6, 56.6, 56.1, 55.0, 50.3, 46.9, 46.1, 42.8, 40.9, 37.0, 28.5, 28.4, 25.5, 25.4, 24.4; HRMS (ESI) *m/z* calcd for C_41_H_43_N_3_O_12_S [M + H]^+^ 802.2646, found 802.2640.

### 2.2. Griess assay

The NO-releasing capacity of each target compound was determined by the colorimetric method under the instructions of the manufacturer. Preparation of standard curves of 0, 1.56, 3.13, 6.25, 12.5, 25, 50 and 100 μmol/L nitrite nitrogen series standard solution was prepared. 50 μL to 96-well plate was added respectively and then mixed with 50 μL Griess I and Griess II; the absorption value was measured at 540 nm wavelength. A standard curve was drawn from the obtained data. Compounds were formulated into 10^−4 mol/l^ with PBS (pH = 7.4) and pharmaceutical DMSO. The newly formulated *L*-cysteine solution 100 μL (3.6 mmol) was added and incubated at 37 °C for 15, 30, 45, 60, 90, 120, 150 and180 min, respectively. Griess I and II recovered to room temperature, 50 μL of compounds **7A–E(a–c)** were added respectively into the 96-well plate, and then 50 μL of Griess I and 50 μL of Griess II were added in sequence, with the absorbance tested at 540 nm. The amount of NO released was then calculated according to the standard curve.

### 2.3. Cck-8 assay

The antiproliferative activities of target compounds were examined by CCK-8 assay in three cancer cells (human lung cancer cell line A549, human mammary gland tumour cell line MCF-7 and human TNBC cell line MDA-MB-231) and one normal cell (human normal breast cell line MCF10A). All cell lines were sourced from KeyGEN Biotech, Beijing, China, and a standard DMEM medium was applied to all cell cultures then subjected to 24 h incubation at 37 °C in a humidified environment with 5% CO_2_. After that logarithmic growing cells were plated on 96-well plates with 8,000 cells per well in a 100 ml medium for 24 h incubation at 37 °C with 5% CO_2_. The DMEM medium with bovine serum was removed from each well. Then, prerequisite target compounds and positive controls were added to different cell lines in a predetermined concentration regiment for 48 h culture. The final step of testing was carried out by adding 10 μL CCK-8 to each well for an hour then shaking for 10 min before the OD value of each well was measured on a Microplate Reader (BioTek Elx800, Winooski, VT) at the wavelength of 450 nm and IC_50_ values were calculated using GraphPad.Prism software with concentration data transformed into logarithms and the inhibition curve was drawn with log(inhibitor) vs. response under variable slope (four parameters) setting.

### 2.4. Cell cycle analysis

This assay was conducted on 6-well plates with 200,000 MDA-MB-231 cells per well and incubated for 24 h at 37 °C with 5% CO_2_, then the medium from each well was removed and treated by **7Cc** in different concentrations (0, 0.41, 0.82, 1.64 μM) for additional 72 h incubation. The desired cell suspension was acquired after attached cells were digested then collected and washed in terms. Cell suspension was then subjected to fixation via 70% ethanol and washed with phosphate-buffered solution (PBS), incubated with 100 μL Rnase A, and placed on water bathed for half an hour at 37 °C. For the final step, 400 μL PI was applied to the mixture in a dark environment at 4 °C for 30 min. Then the distribution of DNA content was measured by flow cytometry (FACS Calibur Becton-Dickinson, Franklin Lake, NJ).

### 2.5. Cell apoptosis assay

This assay was performed on 6-well plates with 200,000 MDA-MB-231 cells per well and incubated for 24 h at 37 °C with 5% CO_2_, then the medium from each well was removed and treated by **7Cc** in different concentrations (0, 0.41, 0.82, 1.64 μM) for additional 72 h incubation. Subsequently, 500 μL of binding buffer was applied to suspend and collect compound treated cells. Then, 5 μL of both annexin V-fluorescein isothiocyanate (Annexin V-FITC) and propidium iodide (PI) was applied to the mixture and this reaction was placed under total dark at room temperature for 10 min. Cell apoptosis was examined by flow cytometry.

### 2.6. Mitochondrial membrane potential assay

Briefly, MDA-MB-231 cells in logarithmic growth were placed in 6-well plates with 200,000 cells per well for 24 h before a medium remove and then incubated with **7Cc** at predetermined concentrations (0, 0.41, 0.82, 1.64 μM) for 48 h. Cells were collected after and washed with PBS. JC-1 dye was added to cells for staining under total dark conditions according to the manufacturer's instructions (KGA601, KeyGEN Biotech, Nanjing, China). The percentage of cells with collapsed mitochondrial membrane potentials was detected by flow cytometry.

### 2.7. Wound healing assay

In this assay, 6-well plates with 200,000 cells per well were used for the culture of MDA-MB-231 cells at 37 °C for 24 h and the medium of each well was removed. Then, **7Cc** at predetermined concentrations (0, 0.41, 0.82, 1.64 μM) was added to the cell culture for incubation. After that, sterile pipette tips were utilised to scratch cell culture in 6-well plates in an even fashion, and detached cells were washed away by PBS with culture medium replaced anew afterward. After 24 h of additional incubation, cell cultures were photographed and migration distance examined accurately.

### 2.8. Transwell assay

24-well transwell plates with 50,000 cells per well were applied to culture MDA-MB-231 cells in their upper chambers with a predetermined concentration of **7Cc** (0, 0.41, 0.82, 1.64 μM) treated cells seeded in the lower surface for 24 h. Then, cells attached to the upper surface of the membrane were removed while migrated or invaded cells of the lower membrane surface were added 0.1% crystal violet staining for 30 min. Then, migrated or invaded cells of the lower chamber were photographed and counted.

### 2.9. Adhesion assay

MDA-MB-231 cells were incubated in different plates and then treated with **7Cc** in different concentrations (0, 0.41, 0.82, 1.64 μM) for 72 h. After that, the CAM (calmodulin) stain treated serum-free medium was applied and 96-well plates were seeded with 100 μL already prepared cell suspension at 2,000 cells per good concentration for an hour. Then, plates were washed by PBS, fixed via 3.7% formaldehyde, another PBS wash, and photographed by fluorescence microscope.

### 2.10. Comet assay

All DNA damage sustained in this assay was measured via Comet Assay Kit (Keygen, Nanjing, China) according to the manufacturer’s instructions. **7Cc** was added to MDA-MB-231 cells at predetermined concentrations (0, 0.41, 0.82, 1.64 μM) for 72 h. These cells were then collected and fixed on CometSlide at 4 °C for 15 min. After that, the cells were set under lysis solution at 4 °C for 90 min and these slides were electrophoresed for 20 min, fixed in ethanol for 5 min, and then stained with Vista Green DNA Dye. The final results were acquired by a fluorescence microscope.

## 3. Results and discussion

### 3.1. Chemistry

**Scheme 1.**
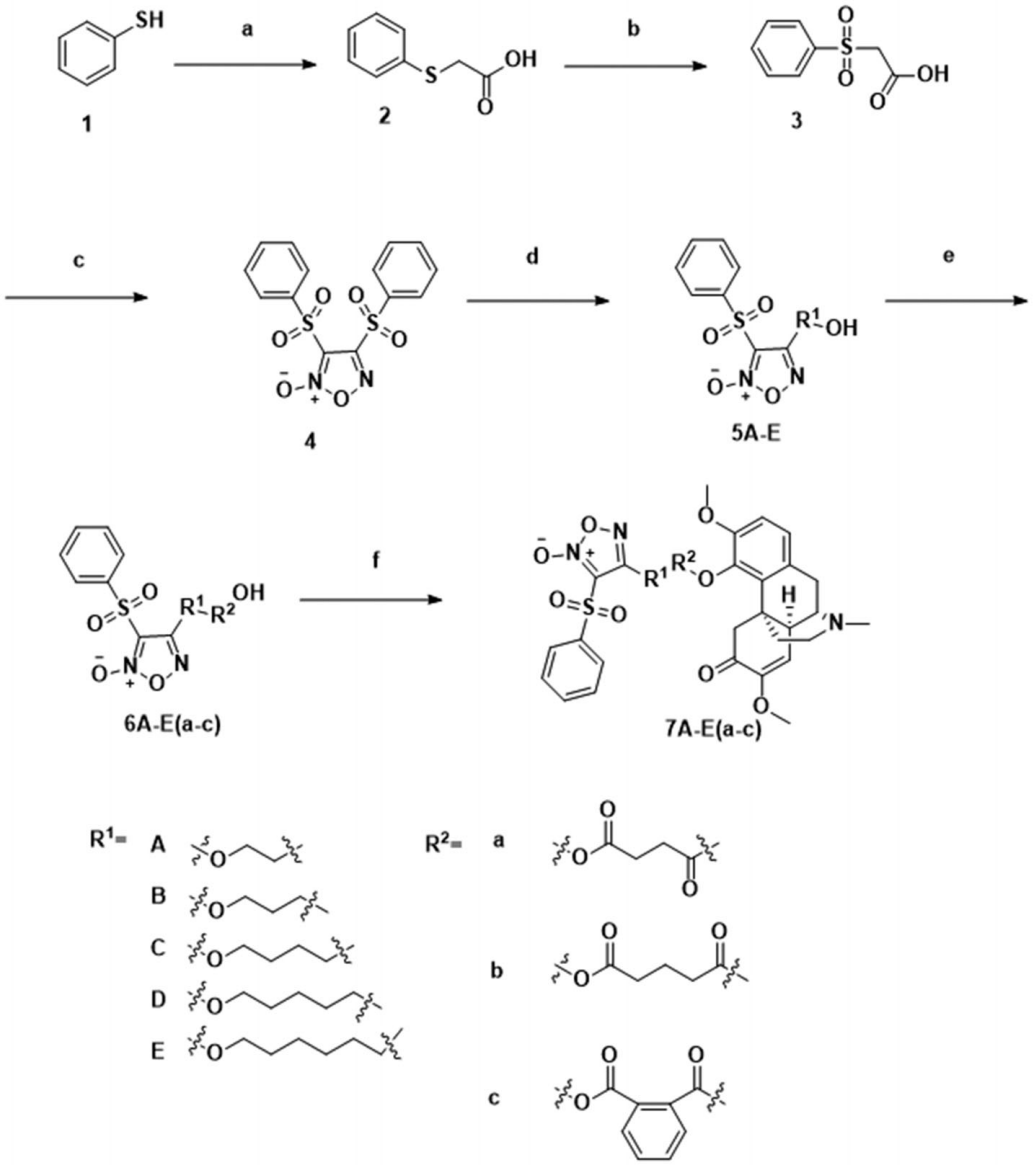
Synthesis of **1**–**4**, **5A**–**E**, **6A**–**E**(**a**–**c**) and **7A**–**E**(**a**–**c**). Reagents and conditions: (a) ClCH_2_COOH, NaOH (aq), reflux, 2 h; (b) 30% H_2_O_2_, AcOH, rt, 3 h; (c) fuming HNO_3_, 100 °C, 8 h; (d) corresponding diol, THF, 30% NaOH, 0 °C, 1 h; (e) corresponding anhydrides, TEA, DMAP, DCM, rt, 2 h; (f) DMAP, EDCI, rt, 4 h.

The synthetic process for compounds **1**–**4**, **5A**–**F**, **6A**–**F**(**a**–**c**) and **7A**–**F**(**a**–**c**) were summarised in Scheme 1. Thiophenol was acidified to 2-phenylsulfanylacetic acid via chloroacetic acid at a high level of heat. Two steps of oxidation subsequently carried out by hydrogen peroxide and fuming nitric acid in room temperature and ice water respectively afforded 1,2,5-oxadiazole with two (phenylsulfonyl)acetic acid fusing together. One phenylsulfonyl group of compound **4** was replaced by a corresponding diol in the alkaline environment under an ice bath for a simple one-step reaction. Intermediary compounds were further extended through the esterification of the corresponding anhydride linked to the hydroxy group in the presence of TEA and DMAP. Target compounds were synthesised by binding sinomenine with intermediary acids **6A**–**F**(**a**–**c**) at room temperature. The structures of target hybrids were confirmed by ^1^H NMR, ^13 ^C NMR and HR-MS.

### 3.2. Biological evaluation

#### 3.2.1. Antiproliferative activity

Target hybrids (**7A**–**E**(**a**–**c**)) and positive controls (cisplatin and adriamycin) were tested for antiproliferative activities against three human cancer cell lines (human mammary gland tumour cell line MCF-7, human lung cancer cell line A549 and TNBC cell line MDA-MB-231), in which evaluation of structure-activity relationship (SAR) and comparison between target compounds and positive controls were carried out. Also, selectivity between normal human cell line MCF10A and two human tumour cell lines (human mammary gland tumour cell line MCF-7 and TNBC cell line MDA-MB-231) were calculated to establish a molecular safety profile.

All tested target compounds exhibited a conspicuous trend of heightened overall antiproliferative effect for *o*-phthalic anhydride linked hybrids while diol linker remained the same. Especially for molecule **7Ac**, benzene ring from *o*-phthalic anhydride led to a steep drop in IC_50_ values to at least one-third of succinic and glutaric linked compounds. The only exception occurred in propane-1,3-diol linked **7B** series hybrids in which *o*-phthalic anhydride **7Bc** showed higher IC_50_ values compared with glutaric anhydride linked one against A549 and MDA-MB-231 cell lines. Extra benzene ring bestowed increased lipophilicity upon sinomenine derivatives, and easier access into tumour cells appeared to facilitate their cytotoxicity to manifest. Although, cytotoxic effect to normal MCF10A cell line also seemed to increase for some *o*-phthalic incorporated Sinomenine derivatives. In terms of diol linker for target hybrids, antiproliferative activities demonstrated comparably low effects in 2–3 carbon-sized chain linked compounds **7A** and **7B** series aside from **7Ac**, almost all cytotoxic results were inferior to that of positive controls (cisplatin and adriamycin). The sole exception was found in **7Bb**, with IC_50_ value of 4.91 μM for A549 cells and just behind a 4.97 μM acquired from cisplatin against the same cell line. From there, as the diol chains lengthened, the cytotoxic effect of 4–5 carbon-linked compounds **7C** and **7D** series were improved as well. IC_50_ values of all 6 hybrids against 3 cancer cell lines stood well below positive controls, with *o*-phthalic incorporated **7Cc** and **7Dc** exhibiting the best effects in the respective series. The length of the diol chain clearly correlated with antiproliferative efficacy closely, while *o*-phthalic incorporated compound **7Cc** still exceeded in **7C** series as in the previous series, but the lowest IC_50_ values observed in **7D** series were more evenly spread among three anhydride types. Bucking the trend, 6 carbon-sized diol linkers in the **7E** compound series failed to continue the upward lift in antiproliferative effect, especially for MCF-7 and MDA-MB-231 cancer cell lines. Although IC_50_ values for the A549 cell line remained below positive control, succinic and glutaric incorporated **7Ea** and **7Eb** demonstrated a reverse in cytotoxicity against MCF-7 and MDA-MB-231 cancer cell lines to a level similar to that of **7A** and **7B** compounds. Yet *o*-phthalic anhydride-linked hybrid **7Ec** seemed to offset part of the decrease in antiproliferative activities and restored cytotoxicity against tumour cell lines back to the level close to **7C** and **7D** series. In addition, the selectivity index of all target hybrids was calculated according to cytotoxicity between two cancer cell lines (MCF-7 and MDA-MB-231) and MCF10A normal human cell line. The results in Table 1 indicated that compound **7Cc** possessed the highest score of 33.57 in SI_MDA-MB-231_, and the second best score of 15.73 in SI_MCF-7_. The aforementioned data clearly showed a combination of potent cytotoxicity against TNBC cell line and high safety differentiation between cancer and normal cell lines for hybrid **7Cc**.

**Table 1.** The antiproliferative effects of the target compounds and parent compounds against different cell lines.

Compound	^a^IC_50_ (*μ*M)					
	A549	MCF-7	MDA-MB-231	MCF-10A	^b^SI_MCF-7_	^c^SI_MDA-MB-231_
**7Aa**	15.25 ± 1.37	26.43 ± 1.25	31.51 ± 2.77	>50	>1.89	>1.58
**7Ab**	9.41 ± 0.68	15.31 ± 0.94	29.58 ± 1.80	>50	>3.27	>1.69
**7Ac**	2.33 ± 0.15	5.87 ± 0.42	1.62 ± 0.15	21.54 ± 0.88	3.67	13.30
**7Ba**	9.84 ± 0.47	18.96 ± 1.33	24.22 ± 1.97	>50	>2.64	>2.06
**7Bb**	4.91 ± 0.30	19.67 ± 1.28	16.45 ± 1.55	>50	>2.54	>3.04
**7Bc**	5.05 ± 0.21	12.18 ± 0.92	18.73 ± 0.61	>50	>4.11	>2.67
**7Ca**	2.97 ± 0.14	2.55 ± 0.14	2.26 ± 0.19	40.68 ± 2.79	15.95	18
**7Cb**	3.53 ± 0.29	4.68 ± 0.39	1.47 ± 0.14	36.71 ± 1.02	7.84	24.97
**7Cc**	1.94 ± 0.05	1.75 ± 0.15	0.82 ± 0.02	27.53 ± 2.34	15.73	33.57
**7Da**	2.79 ± 0.07	1.80 ± 0.11	0.92 ± 0.01	24.74 ± 1.67	13.74	26.89
**7Db**	3.92 ± 0.31	2.94 ± 0.27	3.11 ± 0.22	39.47 ± 2.90	13.43	12.69
**7Dc**	1.63 ± 0.05	2.18 ± 0.10	1.88 ± 0.09	19.85 ± 1.43	9.11	10.56
**7Ea**	4.62 ± 0.16	25.33 ± 0.90	19.00 ± 0.70	43.61 ± 2.65	2.79	2.30
**7Eb**	4.55 ± 0.43	17.89 ± 1.26	41.46 ± 1.48	>50	>2.79	>1.21
**7Ec**	4.81 ± 0.18	3.94 ± 0.28	1.81 ± 0.13	48.32 ± 3.78	12.26	26.70
**Cisplatin**	4.97 ± 0.37	^d^NT	^d^NT	^d^NT	^e^NC	^e^NC
**Adriamycin**	^d^NT	5.12 ± 0.31	4.92 ± 0.44	^d^NT	^e^NC	^e^NC

^a^IC_50_: half inhibitory concentrations measured by the CCK-8 assay. The values are expressed as average ± standard deviation of three independent experiments.

^b^SI_MCF-7_: selectivity index between MCF-7 and MCF-10A. It was calculated as: SI = IC_50(MCF-10A)_/IC_50(MCF-7)_.

^c^SI_MDA-MB-231_: selectivity index between MCF-7 and MDA-MB-231. It was calculated as: SI = IC_50(MCF-10A)_/IC_50(MDA-MB-231)_.

^d^NT: not tested.

^e^NC: not calculated.

Among all the tested hybrids, *cis*-1,4-butanediol and *o*-phthalic anhydride dual-linked sinomenine derivative **7Cc** produced the best cytotoxicity against MCF-7 and MDA-MB-231 cancer cell lines (1.75 and 0.82 μM respectively) with strong antiproliferative effect against A549 behind only **7Dc** (1.94 μM). Hence molecule **7Cc** was most suited for further study into the mode of action for its antiproliferative efficacy against the TNBC cell line.

#### 3.2.2. No release ability

**Figure 2.**
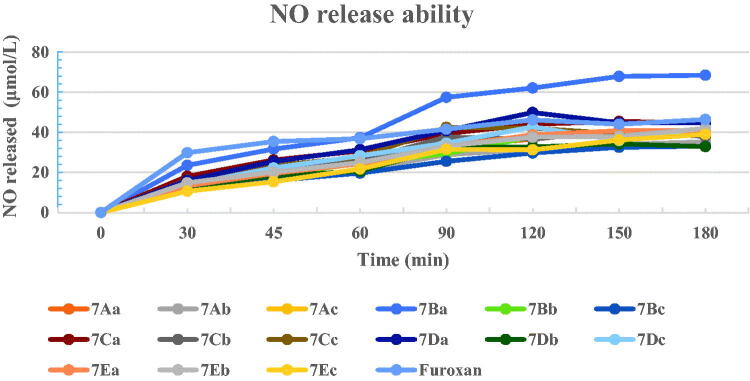
NO-releasing ability of several selected target compounds. The values were expressed as averages of three independent experiments.

As shown in Figure 2, nearly half of all hybrids (**7Aa**, **7Ac**, **7Ba–b**, **7Ca**, **7Da**, **7Dc**, **7Ea–b**) displayed a NO peak releasing exceeding 40 μmol/L, and roughly on the same level as positive control furoxan (46.39 μmol/L). In addition, compound **7Ba** exhibited the highest NO release effect of 68.4 μmol/L at 180 min. Yet the peak amount of NO-releasing appeared not to correlate well with antiproliferative activity against MDA-MB-231 cells. Chiefly among high peak releasing hybrids, only **7Ac**, **7Ca**, **7Da** and **7Dc** possessed excellent cytotoxic effect against all cancer cell lines, other 5 high peak releasing compounds showed lower cytotoxicity than positive control compounds. Intriguingly, the most promising compound **7Cc** produced a NO-releasing speed of only 39.27 μmol/L at 180 min, but reached the top volume of NO released at only 90 min which maintained this level of speed until 120 min and slightly dropped to around 39 μmol/L at 180 min. These results indicated that a relatively fast releasing speed while maintaining that state for a longer period of time might be beneficial to cytotoxic efficacy against cancer cells. A relatively high releasing speed that holds on for a longer period of time might be indicated that the total amount of NO released from **7Cc** could be larger than other higher peak speed releasing ones with a slower start and steep drop from the top. Although NO release only partially represented the cytotoxicity of a target molecule. Other factors might also contribute.

#### 3.2.3. Cell cycle analysis

**Figure 3.**
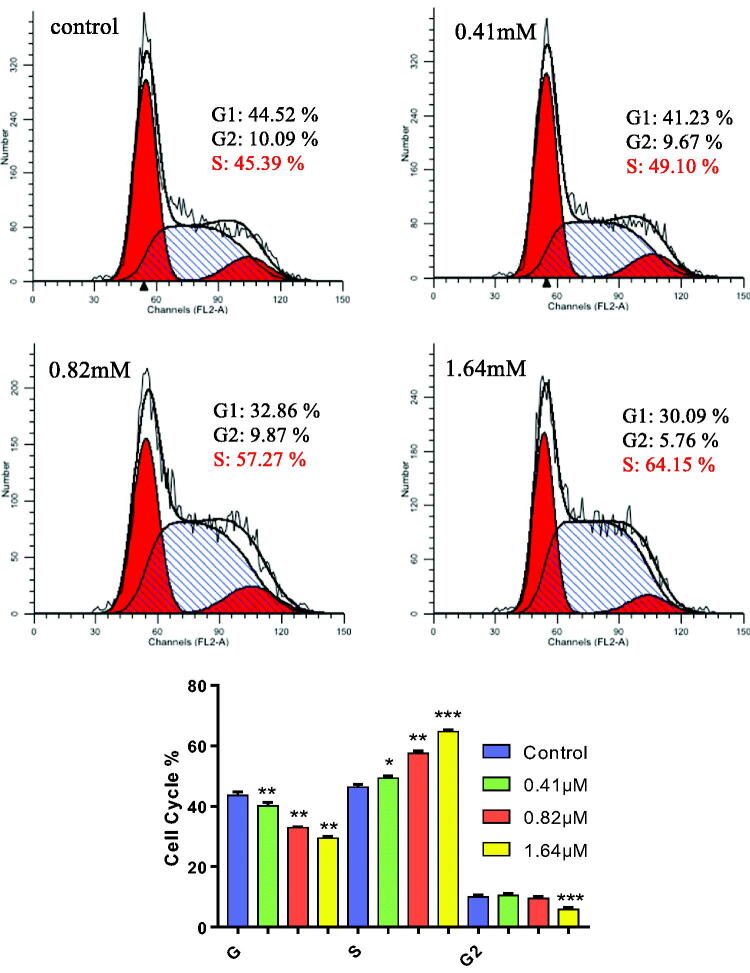
MDA-MB-231 cells were treated with **7Cc** and Cells were stained with PI and investigated by flow cytometry. Data are represented as mean ± SD of three independent experiments. *p < 0.05, **p < 0.01, ***p < 0.001 vs control group. Statistical analyses were carried out on GraphPad.Prism software under ordinary one-way ANOVA method and compared with the control group.

Notable deviation from the normal cell cycle was observed in numerous tumour progression, in which incessant cell proliferation occurred indefinitely. Hence, artificial pause in certain cyclical stages targeted neoplastic cells through chemical treatment constituted a plausible antiproliferative method against cancer, TNBC included. Hybrid **7Cc** in 4 different concentration gradients (0, 0.41, 0.82, 1.64 μM) was examined in MDA-MB-231 cells for the potential effect on cell cycle via PI staining analysis measured on flow cytometry. As indicated in Figure 3, starting from the control group, the rising concentration gradient (1/2 IC_50_, IC_50_ to 2 IC_50_) of **7Cc** coincided with a steady increase of the S phase in the cell cycle progression of MDA-MB-231 cells. In percentage tile terms, the share of the S phase in the cell cycle stood at 45.39% for the control group and increased to 49.10%, 57.27% and 64.15% respectively in a concentration-dependent manner. As to G1 and G2 phases, their share decreased relatively to the S phase, from 44.52% to 30.09% and 10.09% to 5.76% respectively, although the G2 phase stayed roughly unchanged at 1/2 IC_50_ and IC_50_ group. For another indicator, the ratio between G2 and G1 was 1.90 in the control group and then uplifted to 1.94 in all tested groups. These results clearly showed that **7Cc** induced S phase cell cycle arrest in the MDA-MB-231 cell line.

#### 3.2.4. Cell apoptosis assay

**Figure 4.**
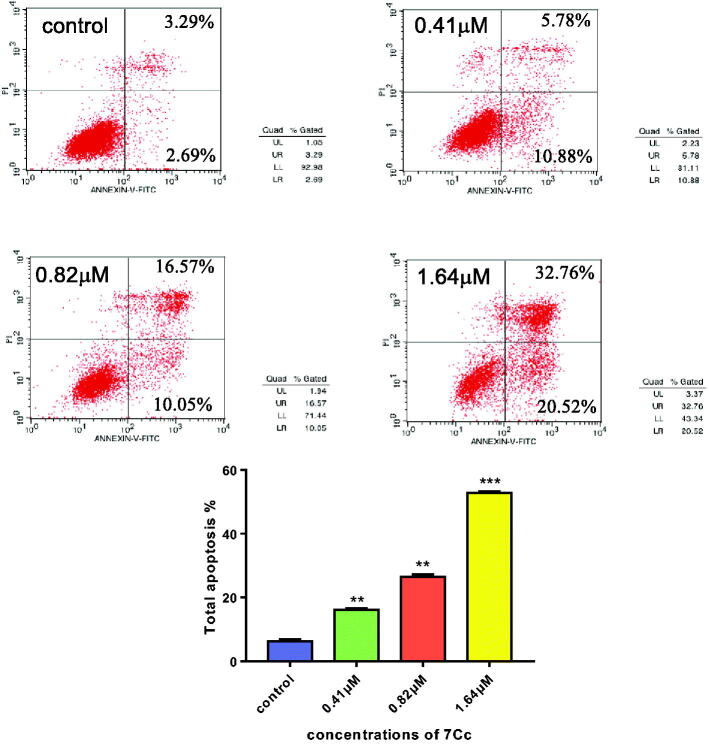
Flow cytometry analysis of apoptosis induced by **7Cc** in MDA-MB-231 cells. Data are represented as mean ± SD of three independent experiments. **p < 0.01, ***p < 0.001 vs control group. Statistical analyses were carried out on GraphPad.Prism software under ordinary one-way ANOVA method and compared with the control group.

Incessant cell growth in tumour without programmatic self-end remained a key component of neoplastic disease, and chemical-induced apoptosis in cancer cells was still considered a pivotal method for tumour treatment. Hence, the apoptotic potential of **7Cc** in the TNBC cell line was examined by the annexin V-FITC/PI binding assay and the results were depicted in Figure 4. The same concentration gradient (0, 0.41, 0.82, 1.64 μM) of **7Cc** was applied in this assay and the percentage shares of apoptosis that occurred in MDA-MB-231 cells were measured in flow cytometry. As Figure 3 indicated, apoptotic portions of cells induced by **7Cc** (two quadrants from the right side) were enhanced from 5.98% of negative control to 16.66, 26.62 and 53.28% with each increased addition of **7Cc** in TNBC cells respectively. This steady induction of apoptosis in MDA-MB-231 cells in a concentration dependant manner affirmed the apoptotic ability of **7Cc**.

#### 3.2.5. Mitochondria membrane potential analysis

**Figure 5.**
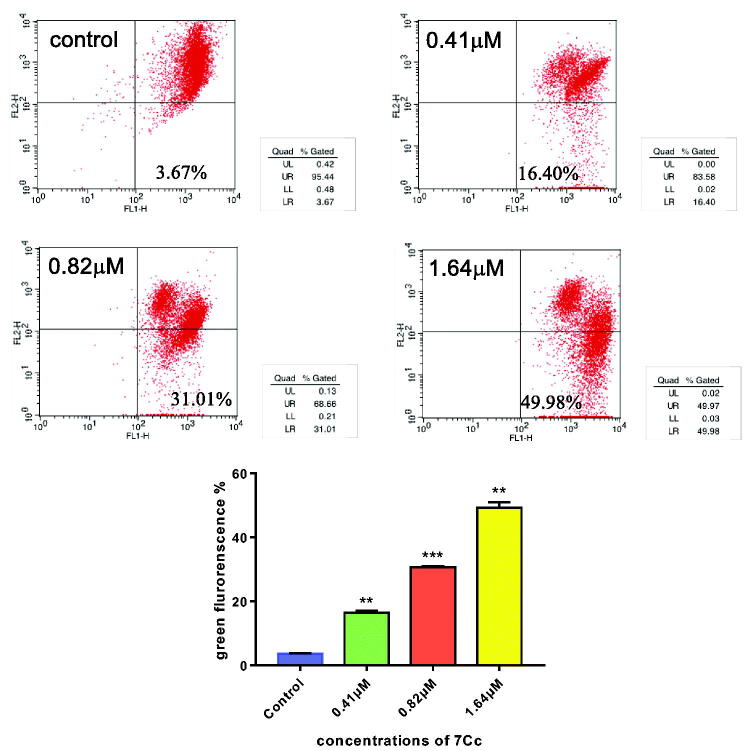
**7Cc** induced mitochondrial depolarisation in MDA-MB-231 cells. Data are represented as mean ± SD of three independent experiments. **p < 0.01, ***p < 0.001 vs control group. Statistical analyses were carried out on GraphPad.Prism software under ordinary one-way ANOVA method and compared with the control group.

Chemical-induced apoptosis in cancer cells could be characterised by abnormal mitochondrial function, which included a slump in mitochondrial membrane potential, a hallmark for drug-facilitated programmed cell death. In an effort to examine whether the mitochondria pathway was utilised in **7Cc** induced TNBC cell apoptosis, the aforementioned concentration gradient (0, 0.41, 0.82, 1.64 μM) was added to MDA-MB-231 cells and the postulated fluctuation in mitochondrial membrane potential was monitored by flow cytometry in a JC-1 staining assay. As displayed in Figure 5, the percentage of apoptotic cells (lower right quadrant) increased from 3.67% of control, to 16.40%, 31.01% and 49.98%, respectively and in a dose-dependent fashion. These results illustrated that **7Cc** caused apoptosis in TNBC cells through intrinsic mitochondrial pathway.

#### 3.2.6. Wound healing assay, transwell assay and adhesion assay

**Figure 6.**
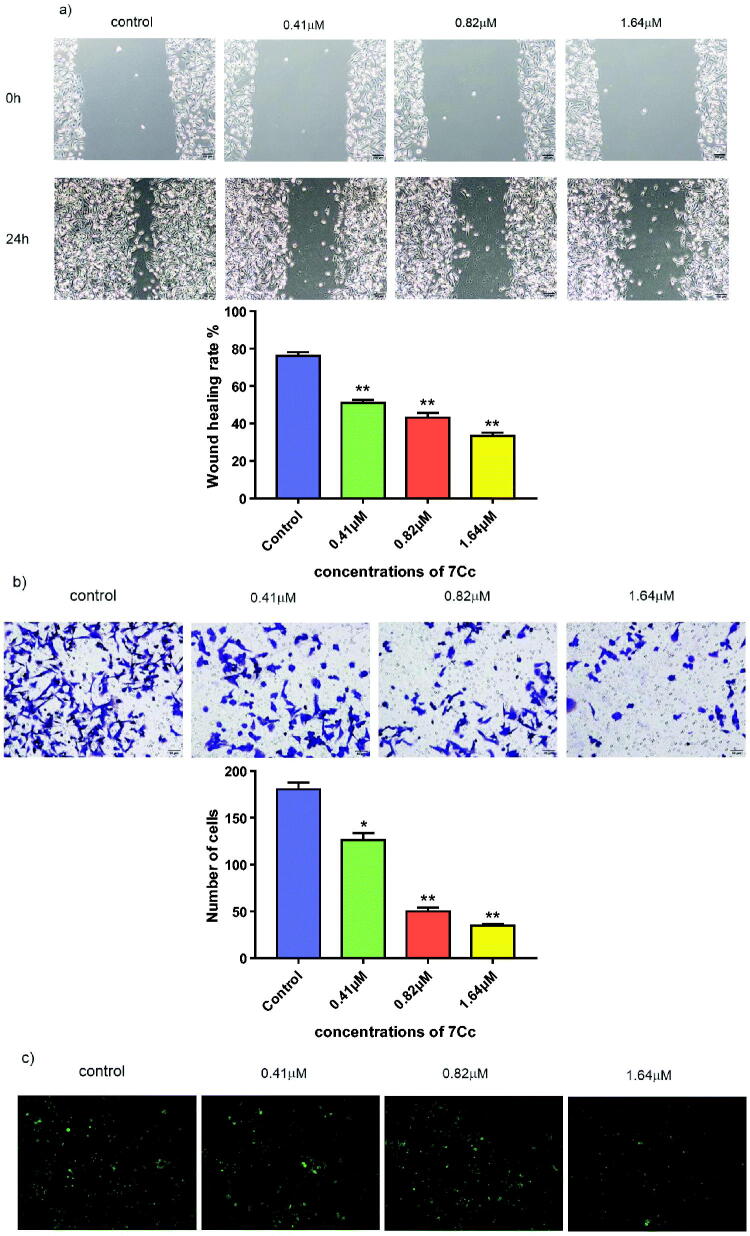
(a) MDA-MB-231 cells were treated with **7Cc**, sterile pipette tips were used to scratch evenly, the incubation was continued, and representative images were captured. (b) MDA-MB-231 cells were seeded onto chambers and incubated with **7Cc**, stained with crystal violet, and representative images were photographed. (c) MDA-MB-231 cells were incubated with **7Cc**, then fixed, washed and photographed with a fluorescence microscope. All data are represented as the mean ± SD of three independent experiments. *p < 0.05, **p < 0.01, ***p < 0.001 vs the control group. Statistical analyses were carried out on GraphPad.Prism software under ordinary one-way ANOVA method and compared with a control group.

The invasive, metastatic feature of tumour growth, TNBC as well, mounted a serious obstacle to anticancer therapy. Inhibitive effects against tumour invasion and metastasis, exerted by medicinal compounds, for example, offered a potential solution. The metastasis-related efficacy of **7Cc** was examined by three consecutive assays as follows. A wound healing assay was performed on MDA-MB-231 cells treated with **7Cc** in four different concentrations (0, 0.41, 0.82, 1.64 μM) and the migratory tendency of each tested group was measured by comparing the distance of wound closure over the same period of time with identical width at the beginning. As shown in Figure 6, after 24 h of incubation, the control group made the most notable advance to close the wound, with only a thin thread remaining. While all three treated groups resisted lateral movement of cancer cells and the wound healed markedly less compared to untreated cells. This result indicated that **7Cc** inhibited migration in MDA-MB-231 cells. Transwell assay was performed on MDA-MB-231 cells with the same treatment of **7Cc** and the invasive activity of TNBC cells along with the inhibitive effect of target hybrid were evaluated. As illustrated in Figure 5, the presence of **7Cc** notably reduced the number of cells permeated through the membrane in the transwell chamber and the decreased invasive ability of TNBC cells was correlated with an increase in hybrid concentration applied to each individual group. The adhesion assay was carried out on MDA-MB-231 cells to evaluate the ability of **7Cc** to detach cancer cells away from each other and prevent cellular adhesion that facilitated metastasis and invasion. The results in Figure 5 exhibited clear prevention of TNBC cellular adhesion in **7Cc** treated groups compared to control and this adhesive inhibition was conducted in a dose-dependent manner. Hence, the aforementioned assays demonstrated the inhibitive effects of **7Cc** on MDA-MB-231 cells in both migration, invasion and adhesion.

#### 3.2.7. Comet assay

**Figure 7.**
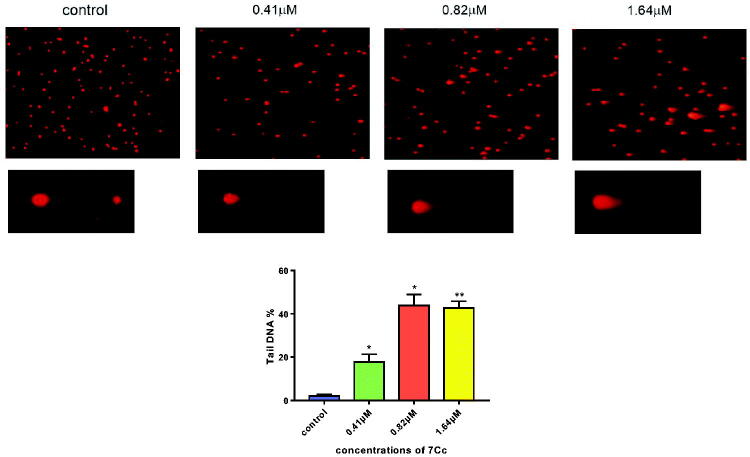
MDA-MB-231 cells were incubated with **7Cc**. Comet assay was used to evaluate DNA damage and photomicrographs were provided. Data are represented as mean ± SD of three independent experiments. *p < 0.05, **p < 0.01 vs control group. Statistical analyses were carried out on GraphPad.Prism software under ordinary one-way ANOVA method and compared with a control group.

Chemical-induced apoptosis in cancer cells was characterised by DNA fragmentation to lesser fractures. The excess genotoxic stress released by neoplastic molecules was considered an effective way of combating tumour malignancy, albeit often came with a serious adverse effect. Therein, **7Cc** has examined for a potential DNA detrimental ability against TNBC cells while maintaining a relatively wide therapeutic window and a standard neutral comet assay was employed to measure double-strand DNA breaks (DSBs) adducts (the tail of a comet) which correlated with the degree of DNA damage. As depicted in Figure 7, the significant presence of endogenous DSBs was observed in the image after the application of **7Cc**. And the share of DNA accumulated in the comet tail was lifted up concurrently with the increased level of **7Cc** delivered. In the meantime, the head of the comet saw its size drop accordingly. This migratory movement of either slim or Olive size after **7Cc** treatment would be a robust testament to notable inhibition of DNA replication and detrimental effect on DNA integrity, especially in a dose-dependent manner. Although an increased level of **7Cc** applied lowered the share of tail DNA, the recorded significant difference was uplifted to signal better reliability. These results indicated that **7Cc** induced apoptosis in MDA-MB-231 cells partially through stimulating DNA damage and halting DNA replication.

## 4. Conclusions

In this article, a series of sinomenine/furoxan hybrid compounds were designed and synthesised. The antiproliferative potential of all target molecules was screened by three human cancer cell lines (human mammary gland tumour cell line MCF-7, human lung cancer cell line A549 and TNBC cell line MDA-MB-231) and one human normal cell line MCF10A. The results showed that all tested compounds exhibited an antiproliferative effect, in which more superior efficacy than positive controls was observed among some derivatives, in one or all three cancer cell lines. These compounds were also holding a relatively high therapeutic window. Of all target derivatives, hybrid **7Cc** produced the best cytotoxicity against MCF-7 and MDA-MB-231 cancer cell lines (1.75 and 0.82 μM respectively) with the second highest effect against A549 (1.94 *μ*M). In addition, **7Cc** obtained the highest SI value for both MCF-7 and MDA-MB-231 cells in comparison with MCF10A cells, which made it the safest derivative among all compounds. Later studies for the mode of action established that **7Cc** induced S phase cell cycle arrest in MDA-MB-231 cells, stimulated apoptosis in MDA-MB-231 cells, disrupted mitochondrial membrane potential and exerted a genotoxic effect on DNA. In separate assays, **7Cc** also notably inhibited MDA-MB-231 cells in both migration, invasion and adhesion. The aforementioned studies demonstrated a strong antiproliferative efficacy of **7Cc** against TNBC cells, and as a promising drug candidate for potential breast cancer therapy, this molecule warranted further research.

## Supplementary Material

Supplemental Material
