# Supplementary material for: Anti-breast cancer sinomenine derivatives via mechanisms of apoptosis induction and metastasis reduction
Source: J Enzyme Inhib Med Chem. 2022 Jul 8;37(1):1870–83. doi: 10.1080/14756366.2022.2096020 (PMC9272937; doi:10.1080/14756366.2022.2096020)
Supplement: Supplemental Material [file IENZ_A_2096020_SM6828.pdf]

Anti-breast cancer sinomenine derivatives via mechanisms of apoptosis induction and metastasis reduction

Xiang Gao[a], Baojia Sun[b], Yonglian Hou[a], Lilin Liu[a], Jianan Sun[a], Fanxing Xu[c], Dahong Li[a,*], Huiming Hua[a,*]

[a]*Key Laboratory of Structure-Based Drug Design & Discovery, Ministry of Education, and School of Traditional Chinese Materia Medica, Shenyang Pharmaceutical University, 103 Wenhua Road, Shenyang 110016, P. R. China*

[b]*Yantai Valiant Pharmaceutical Co. Ltd., 60 Taiyuan Road, Dajijia Industrial Park, YEDA Yantai, Shandong 264006, PR China*

[c]*Wuya College of Innovation, Shenyang Pharmaceutical University, 103 Wenhua Road, Shenyang 110016, PR China*

[*]Corresponding author. E-mail address: lidahong0203@163.com (D. Li); huimhua@163.com (H. Hua)

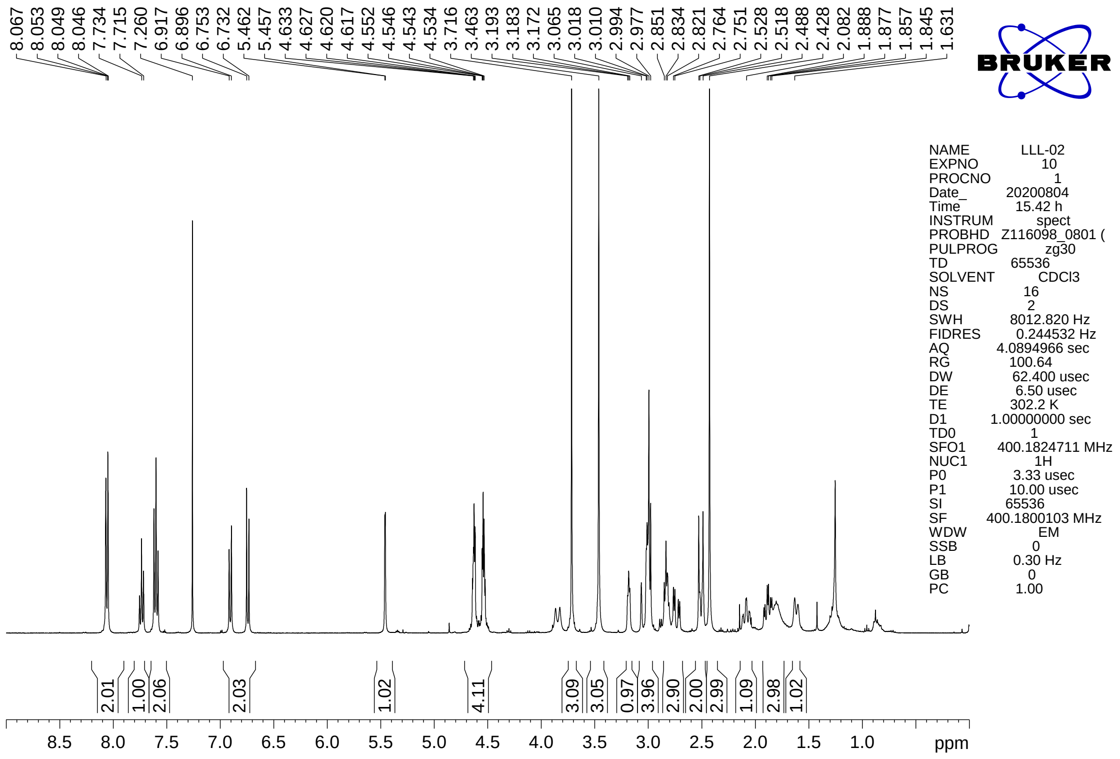

Figure S1. ^{1}H NMR of compound **7Aa**

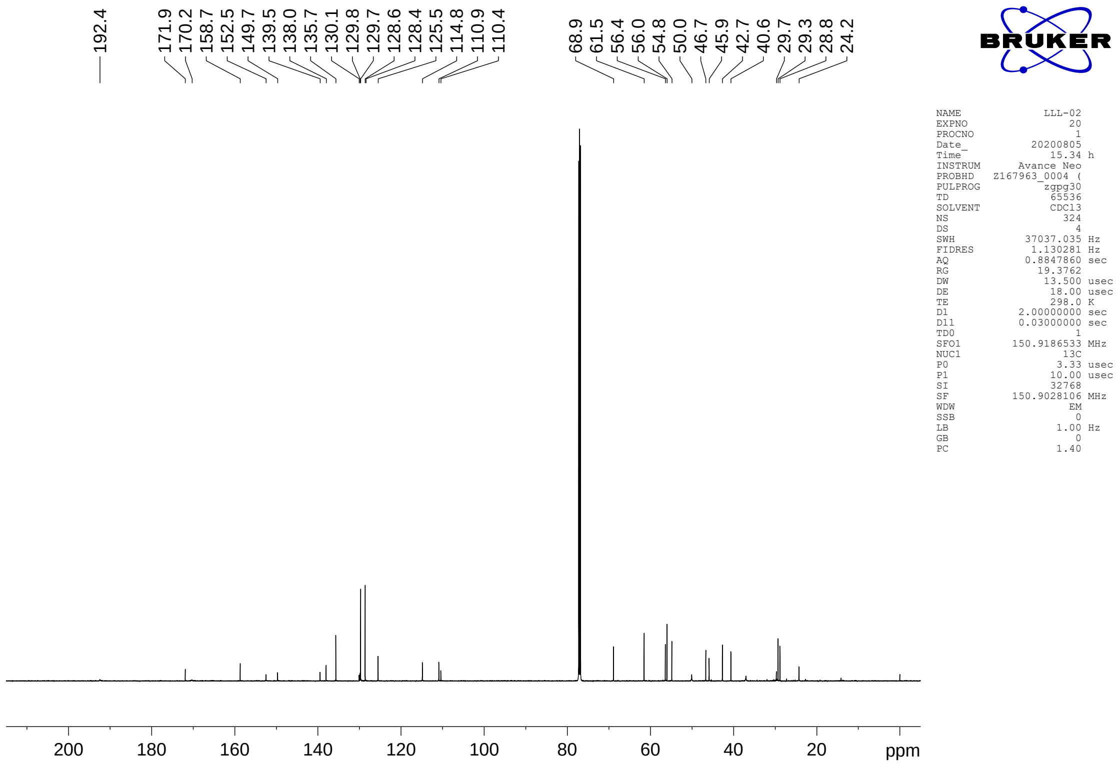

Figure S2. ^{13}C NMR of compound **7Aa**

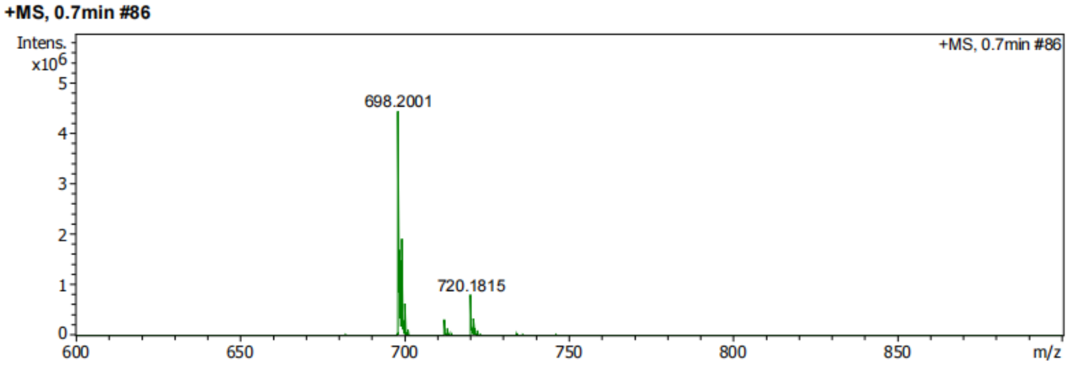

Figure S3. HRESIMS of compound **7Aa**

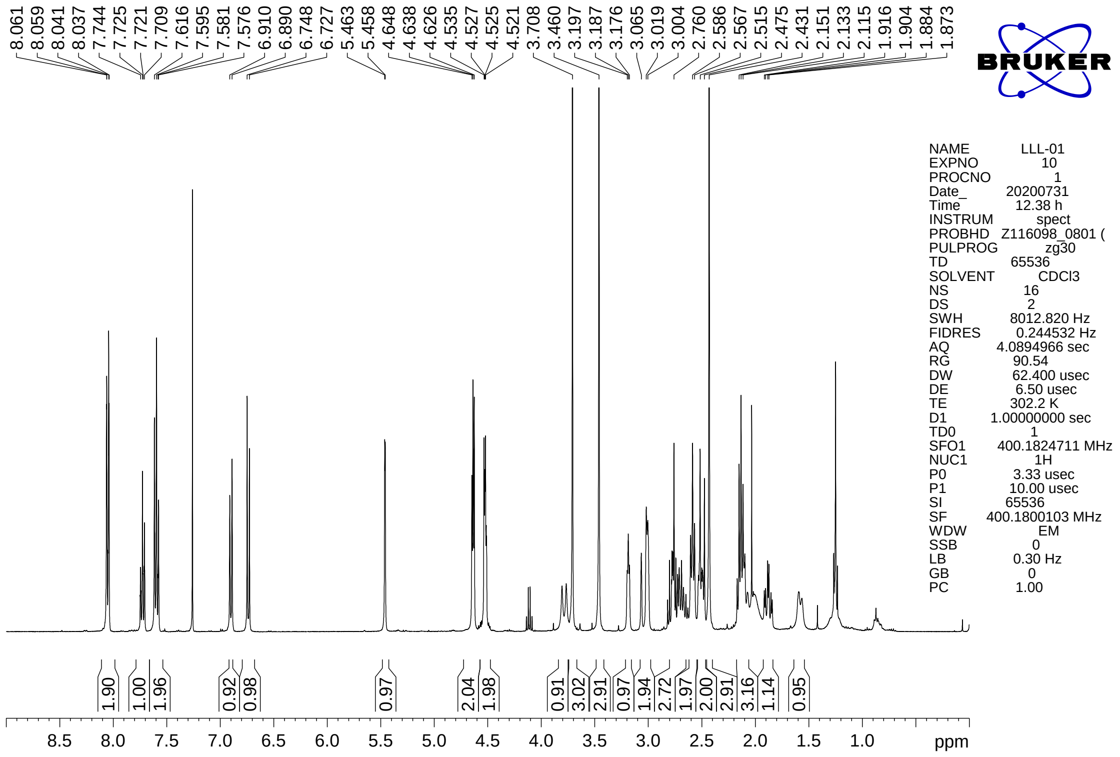

Figure S4. ^{1}H NMR of compound **7Ab**

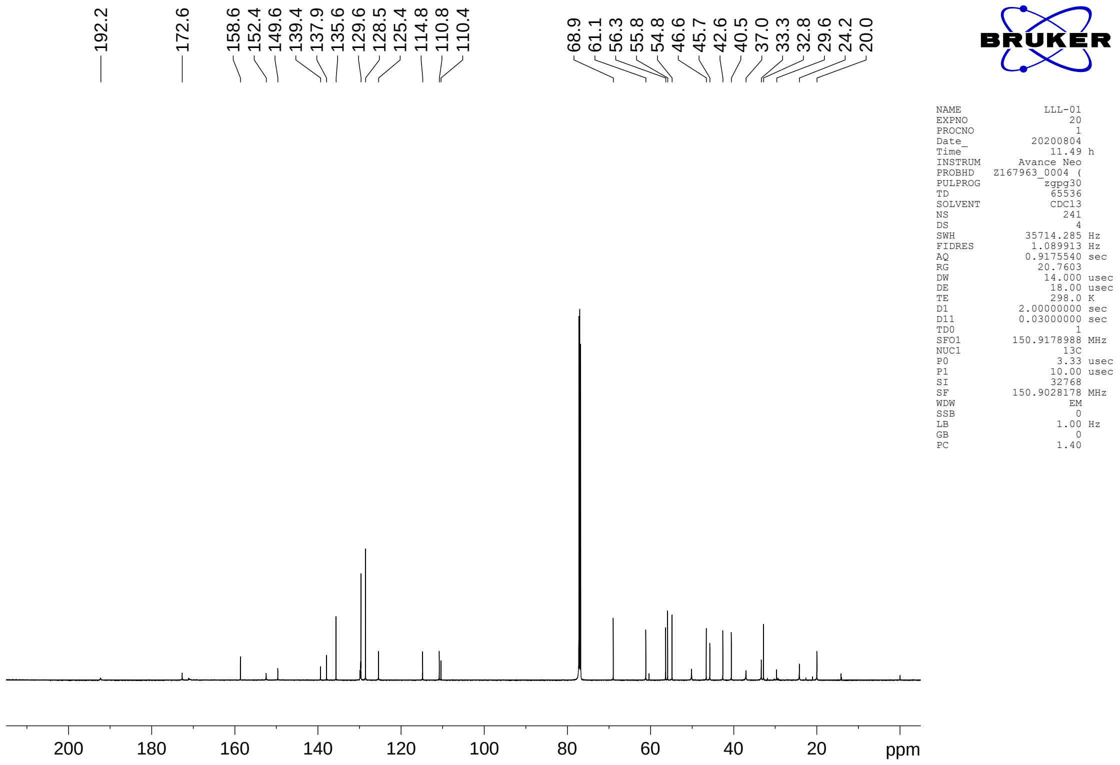

Figure S5. ^{13}C NMR of compound **7Ab**

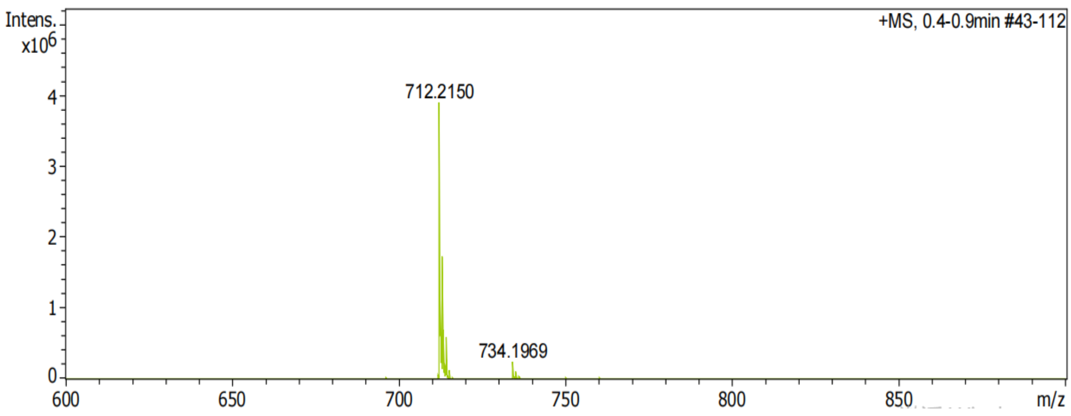

Figure S6. HRESIMS of compound **7Ab**

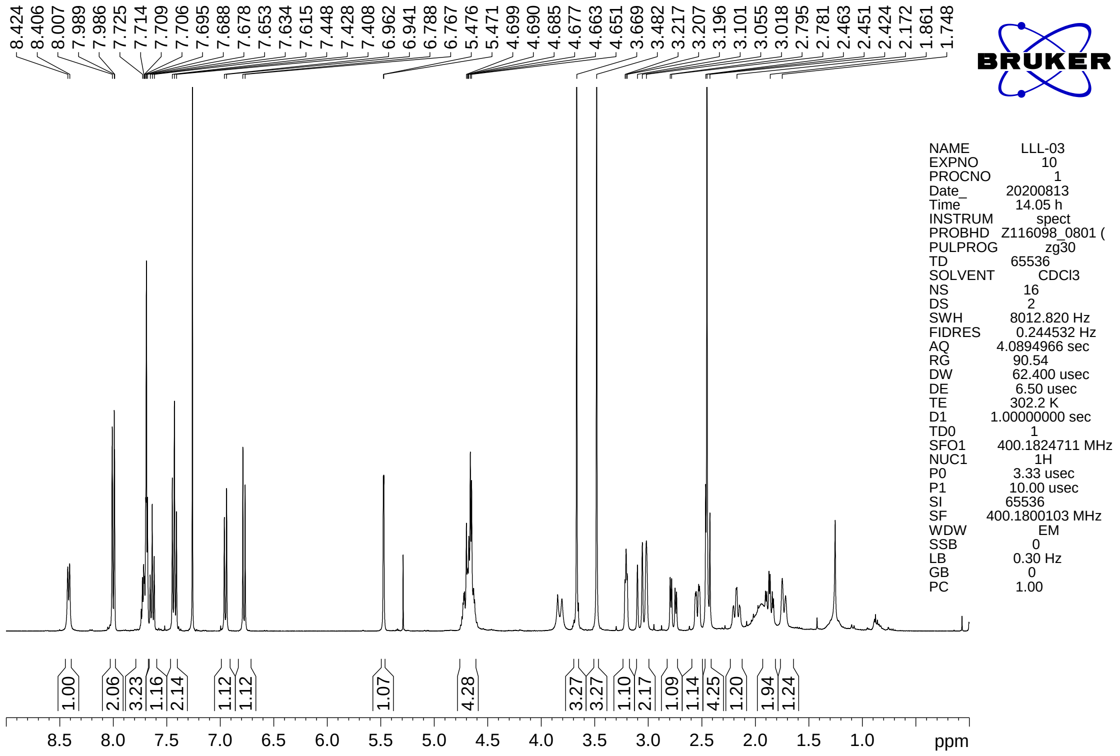

Figure S7. ^{1}H NMR of compound **7Ac**

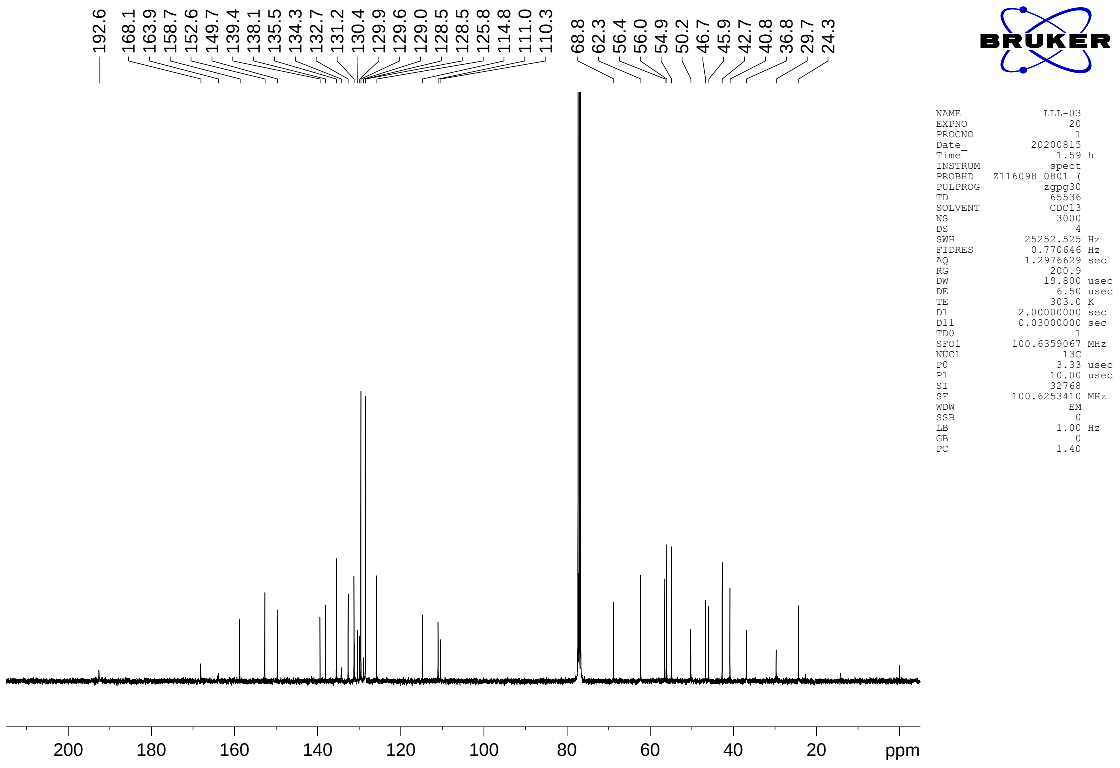

Figure S8. ^{13}C NMR of compound **7Ac**

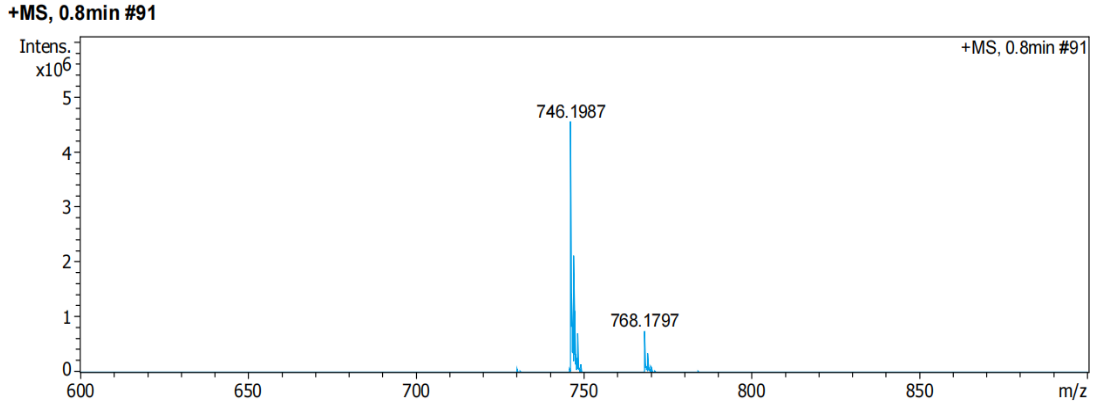

Figure S9. HRESIMS of compound **7Ac**

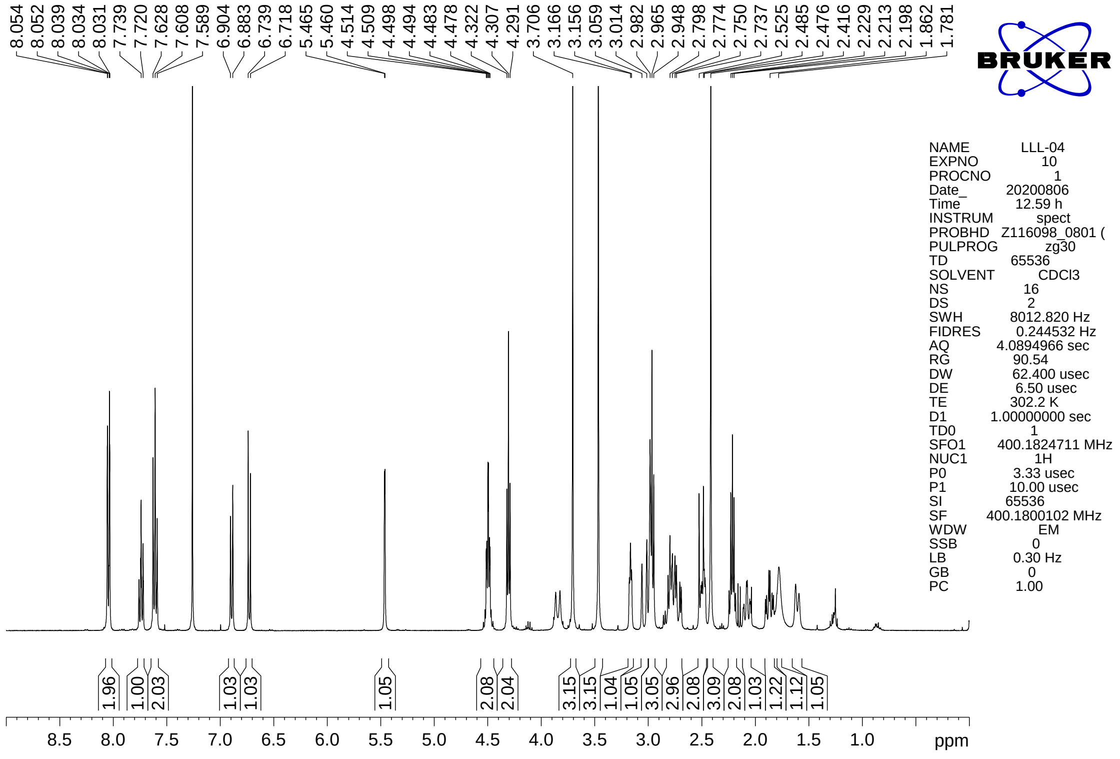

Figure S10. ^{1}H NMR of compound **7Ba**

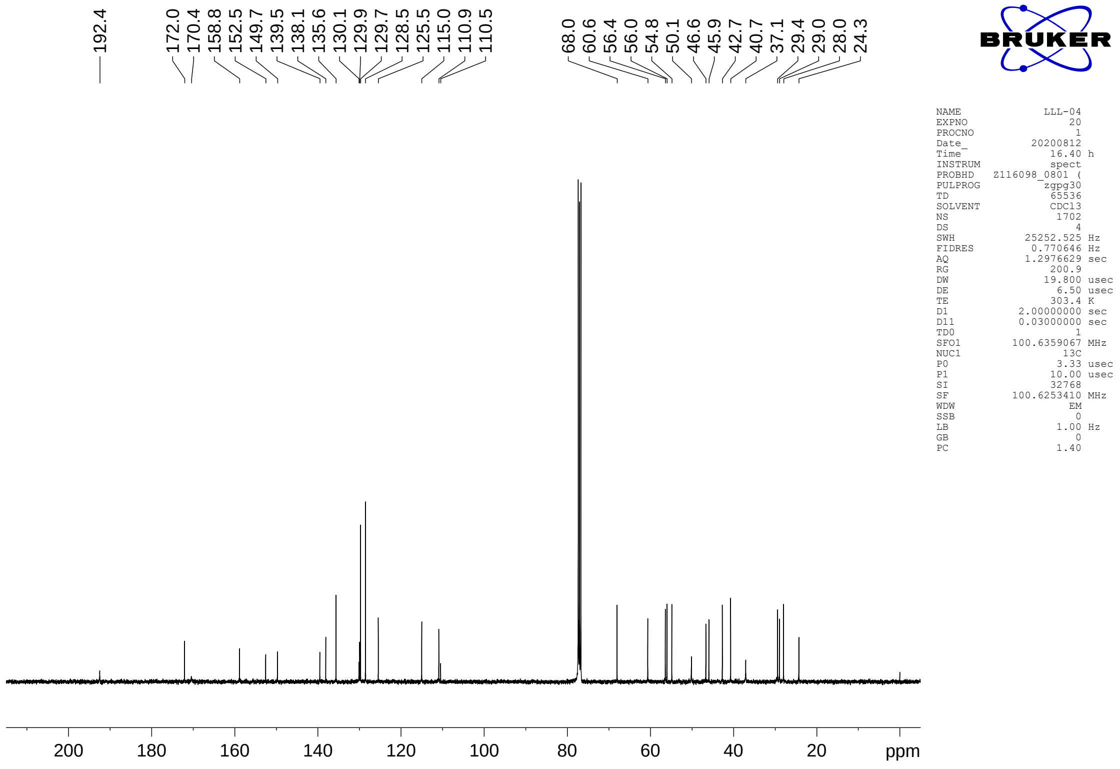

Figure S11. ^{13}C NMR of compound **7Ba**

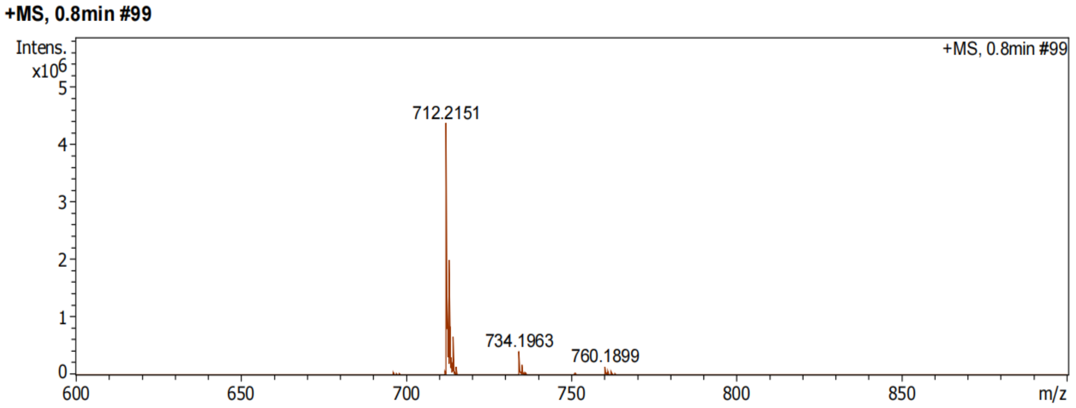

Figure S12. HRESIMS of compound **7Ba**

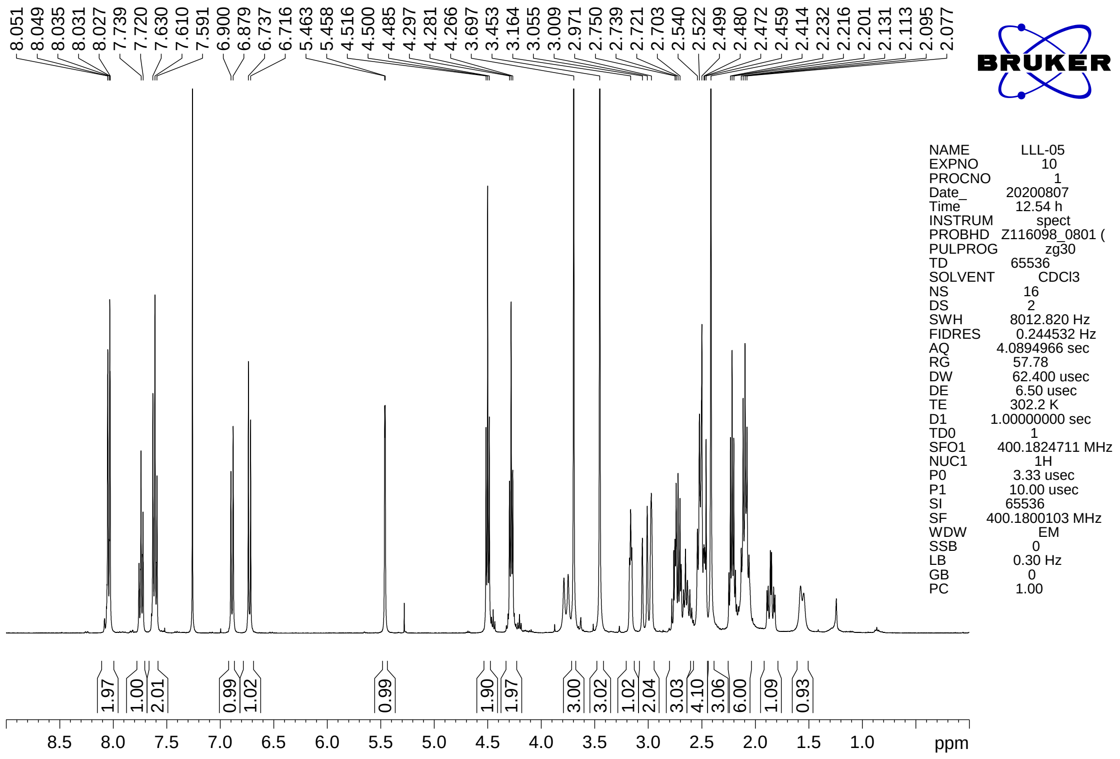

Figure S13. ^{1}H NMR of compound **7Bb**

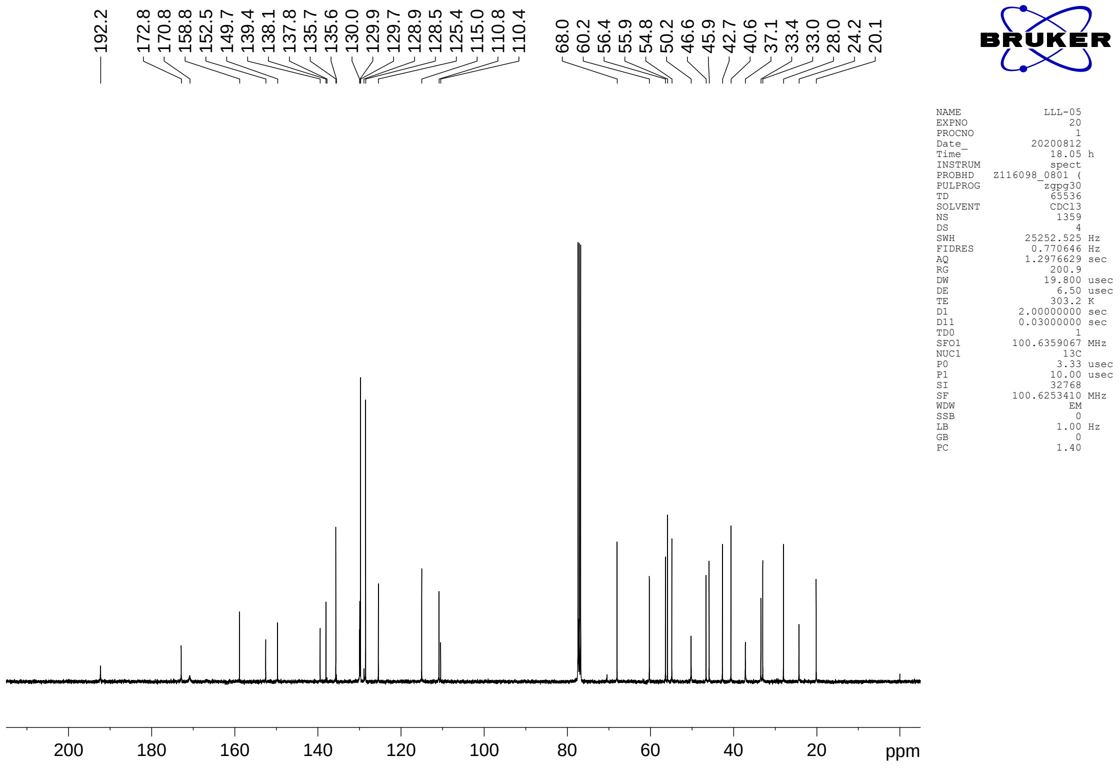

Figure S14. ^{13}C NMR of compound **7Bb**

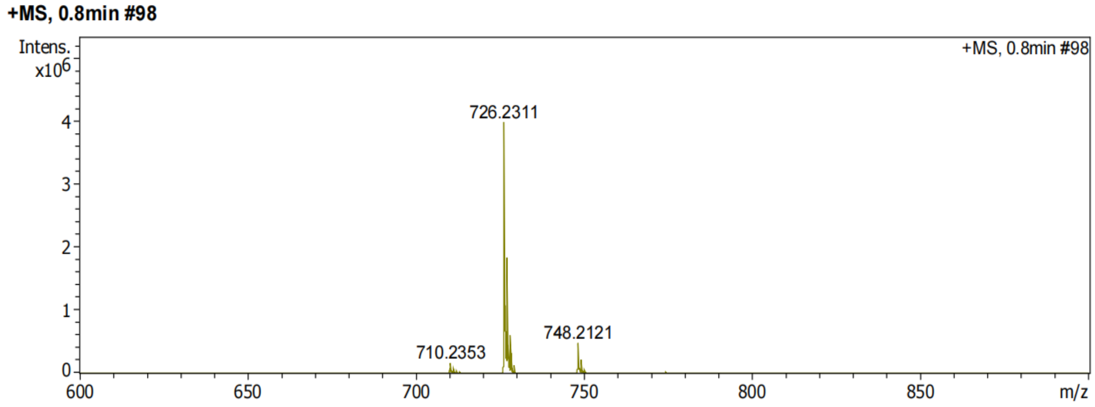

Figure S15. HRESIMS of compound **7Bb**

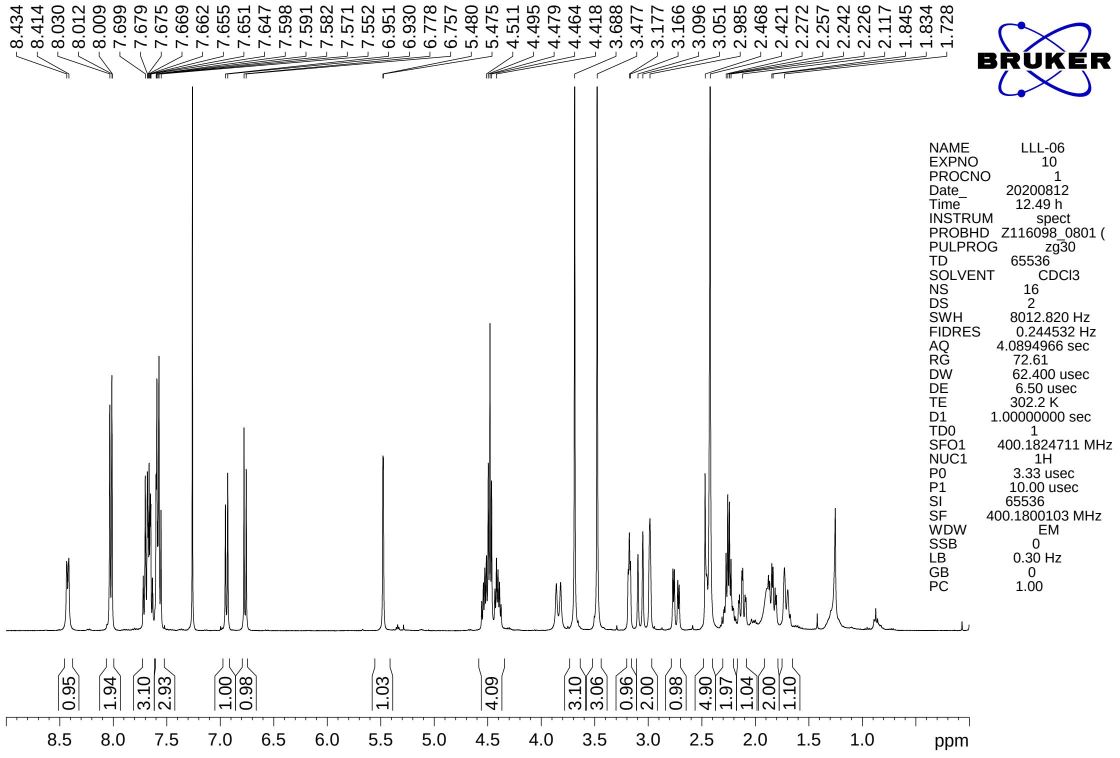

Figure S16. ^{1}H NMR of compound **7Bc**

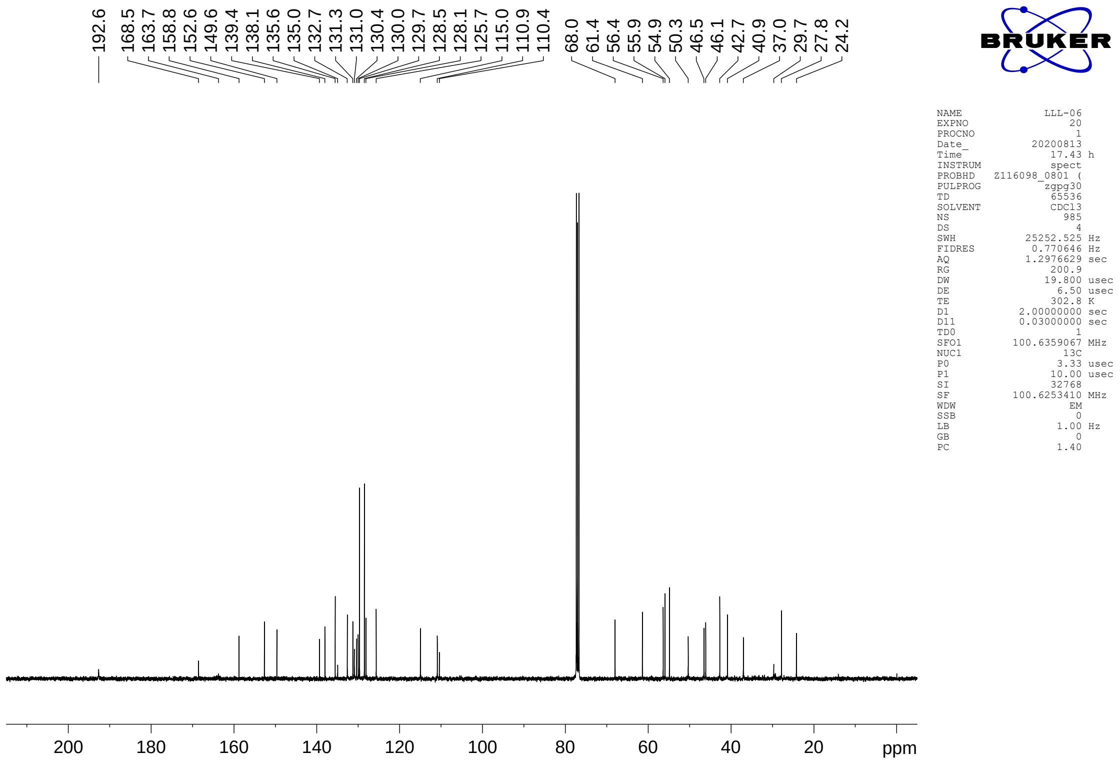

Figure S17. ^{13}C NMR of compound **7Bc**

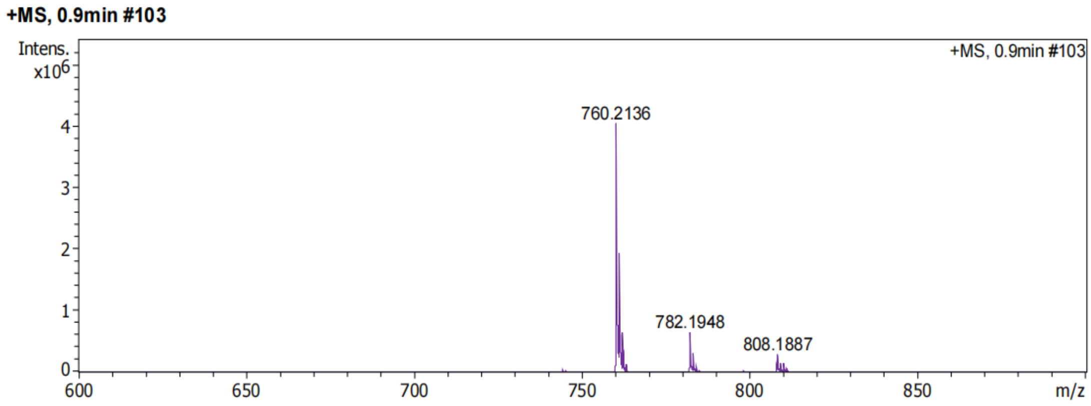

Figure S18. HRESIMS of compound **7Bc**

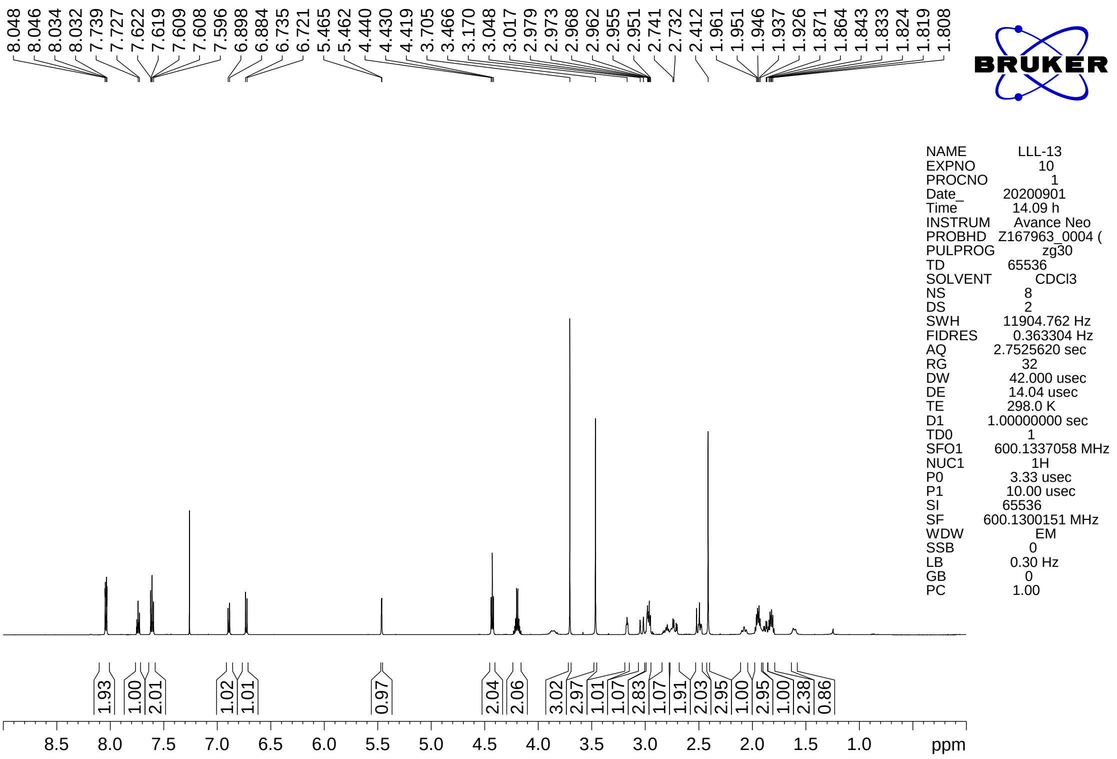

Figure S19. ^{1}H NMR of compound **7Ca**

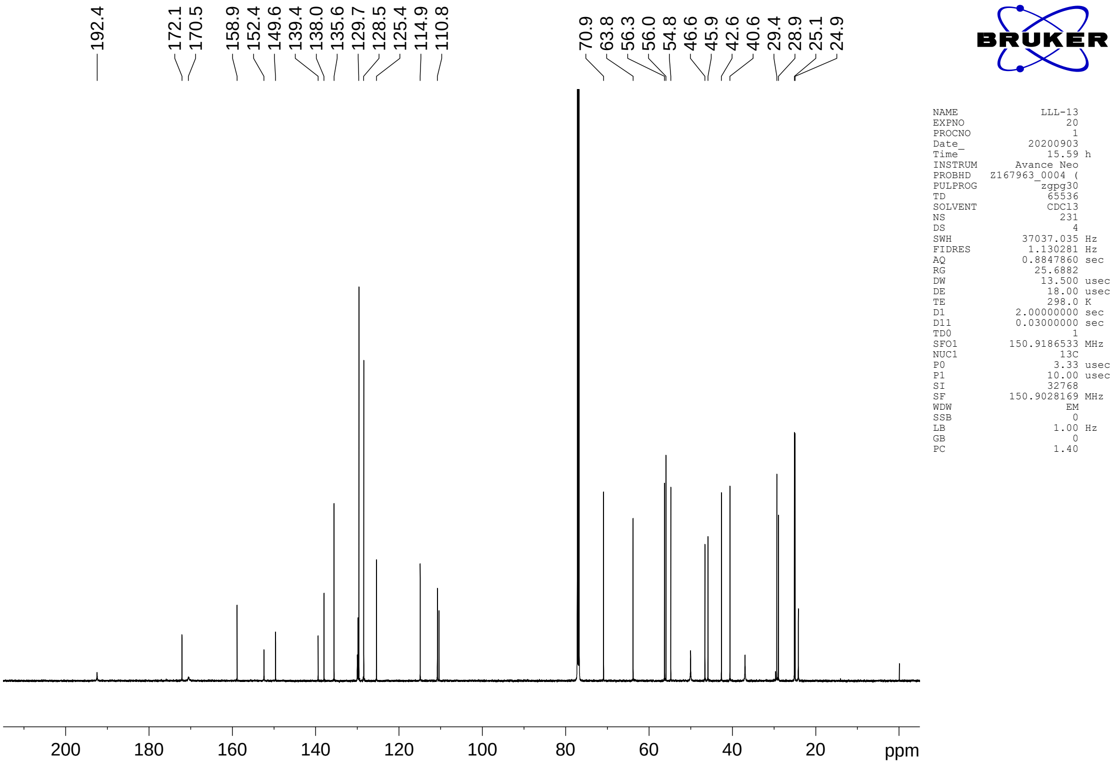

Figure S20. ^{13}C NMR of compound **7Ca**

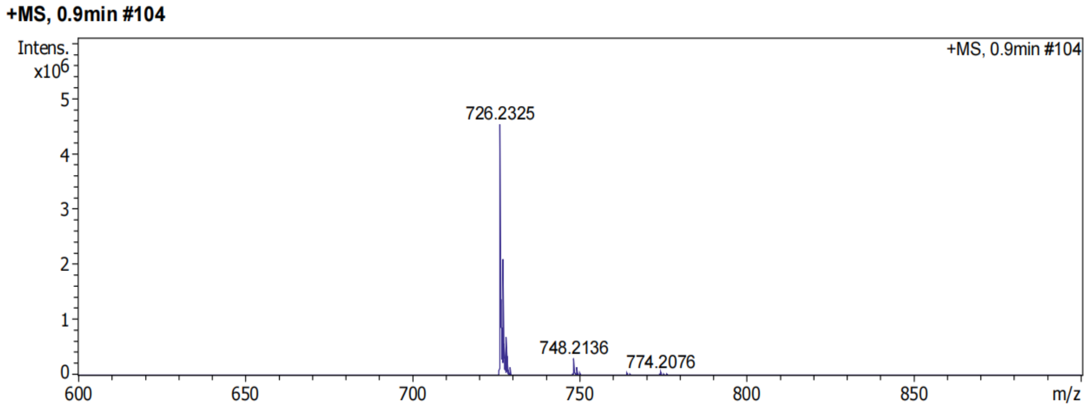

Figure S21. HRESIMS of compound **7Ca**

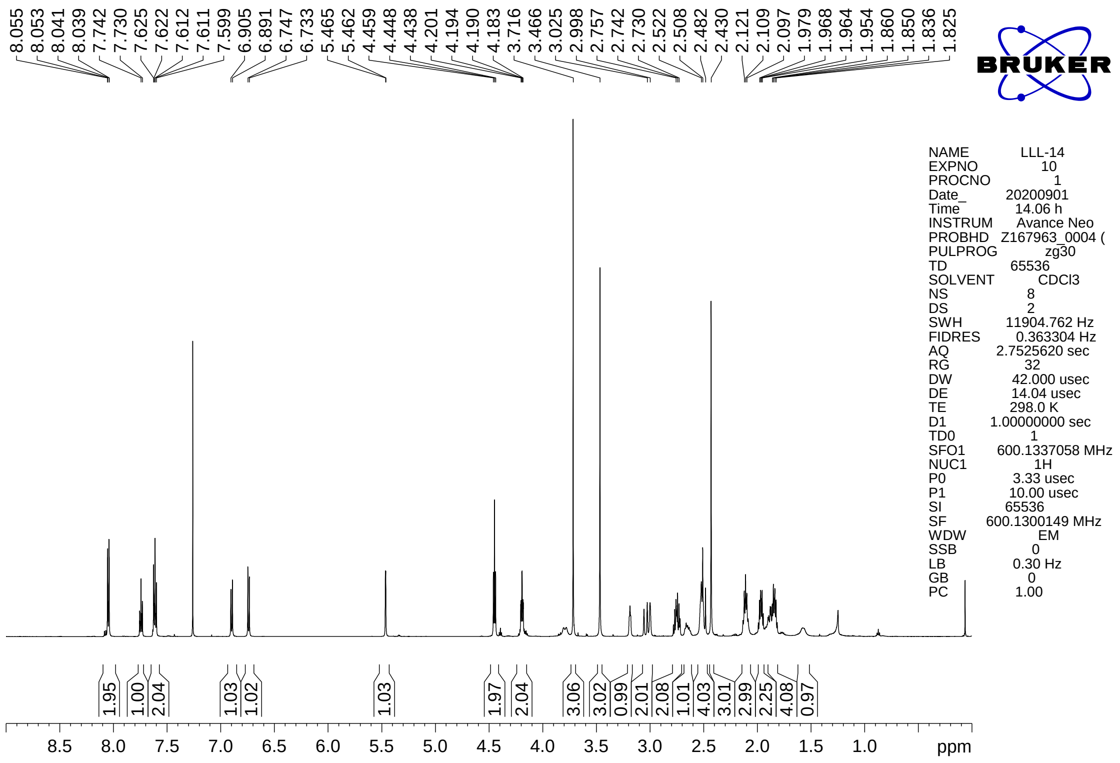

Figure S22. ^{1}H NMR of compound **7Cb**

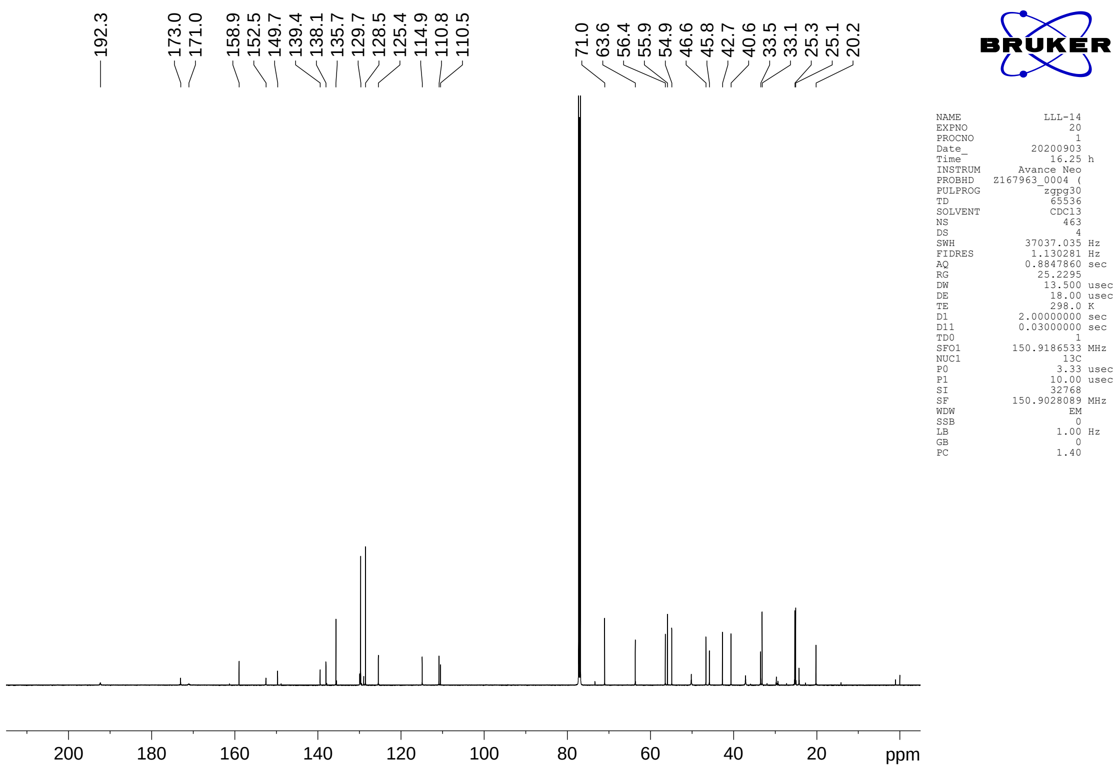

Figure S23. ^{13}C NMR of compound **7Cb**

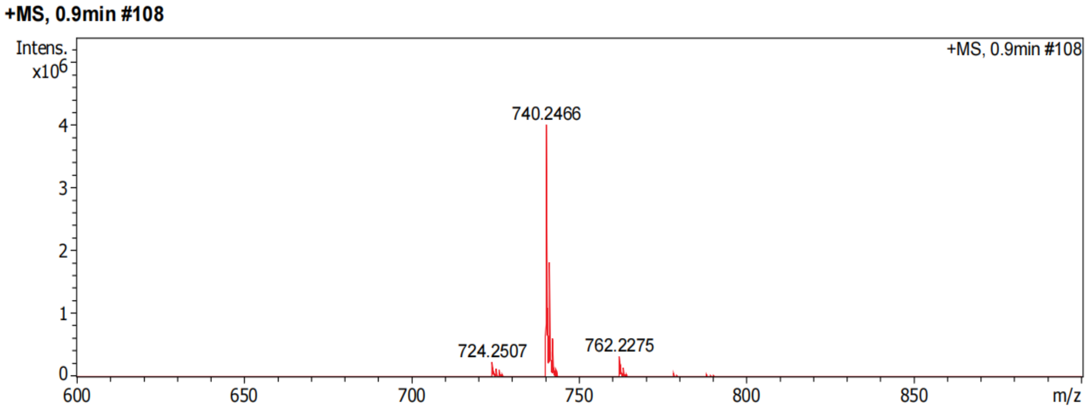

Figure S24. HRESIMS of compound **7Cb**

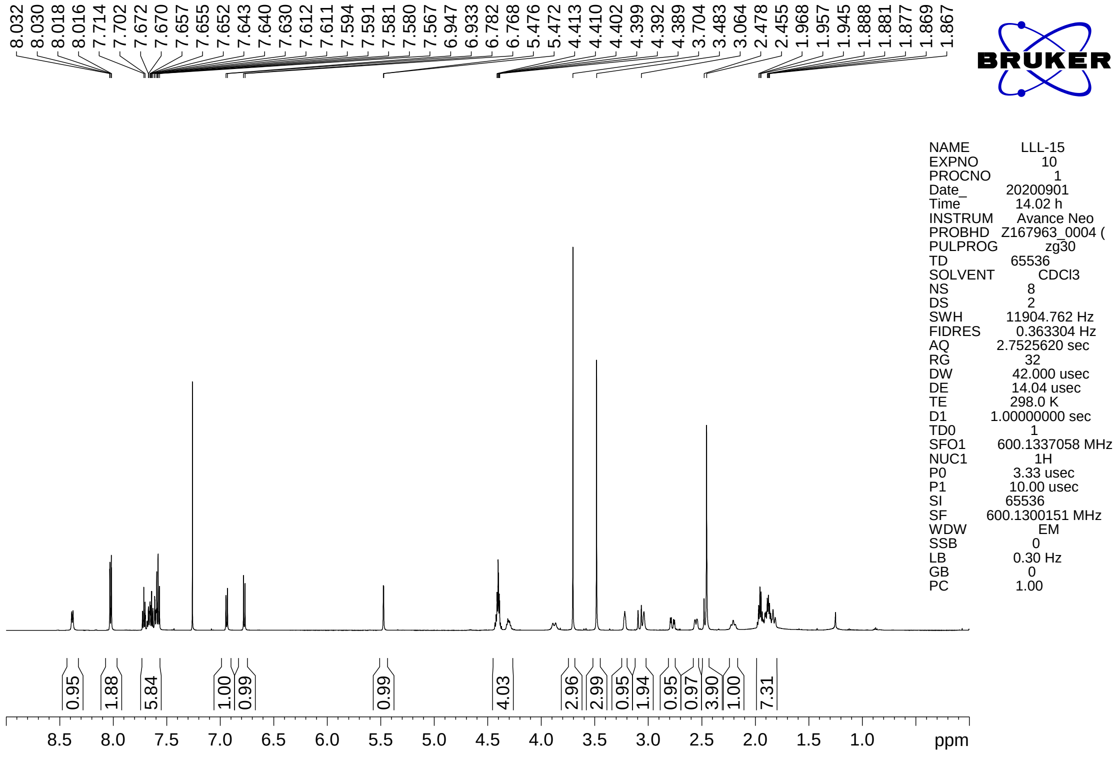

Figure S25. ^{1}H NMR of compound **7Cc**

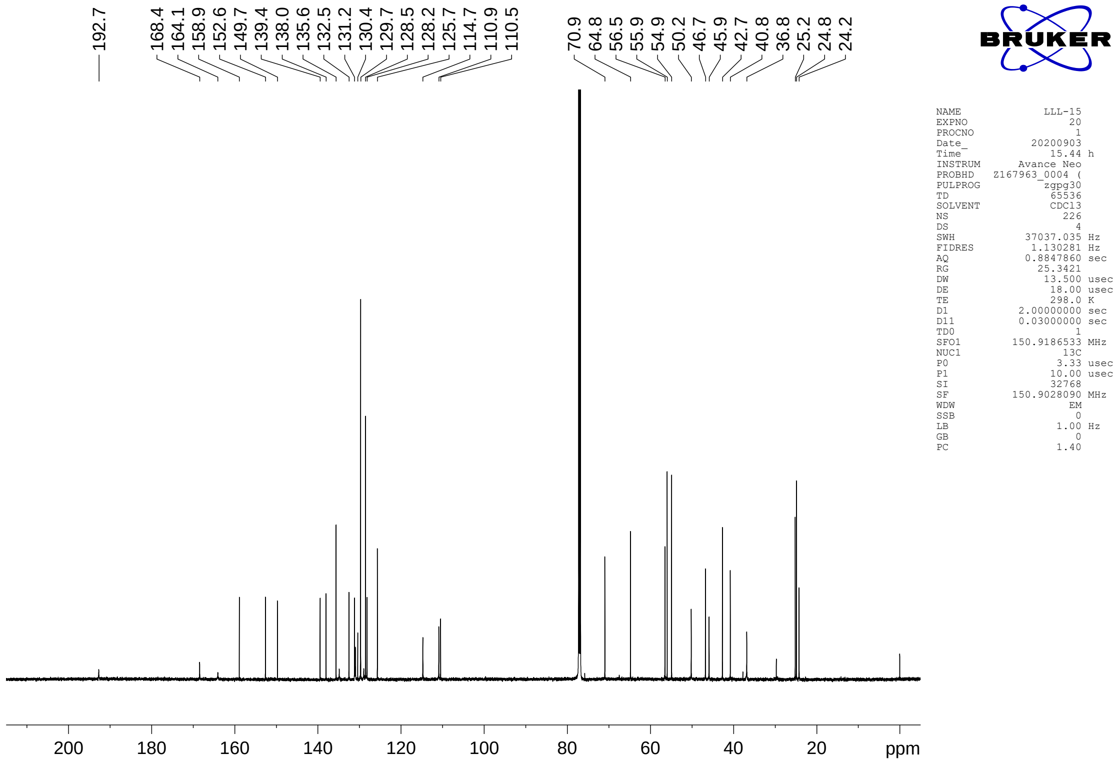

Figure S26. ^{13}C NMR of compound **7Cc**

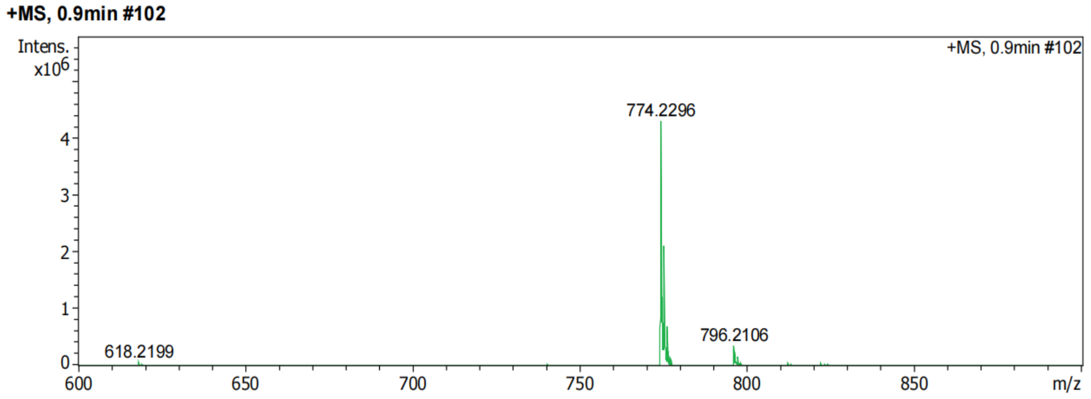

Figure S27. HRESIMS of compound **7Cc**

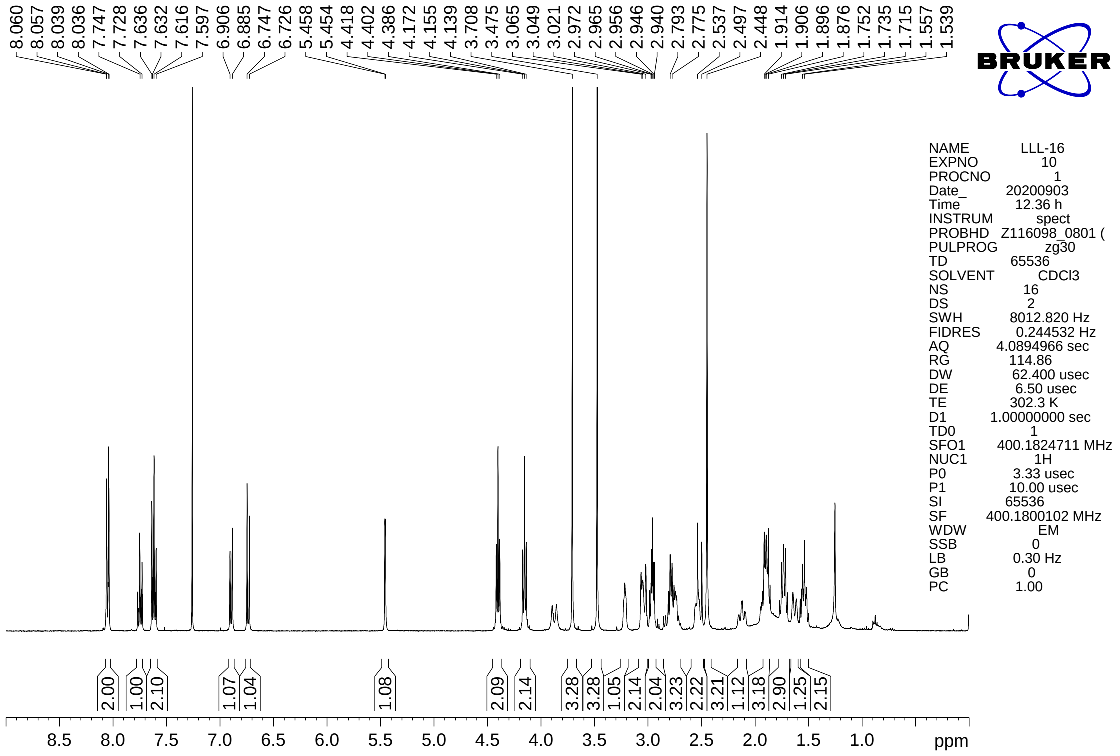

Figure S28. ^{1}H NMR of compound **7Da**

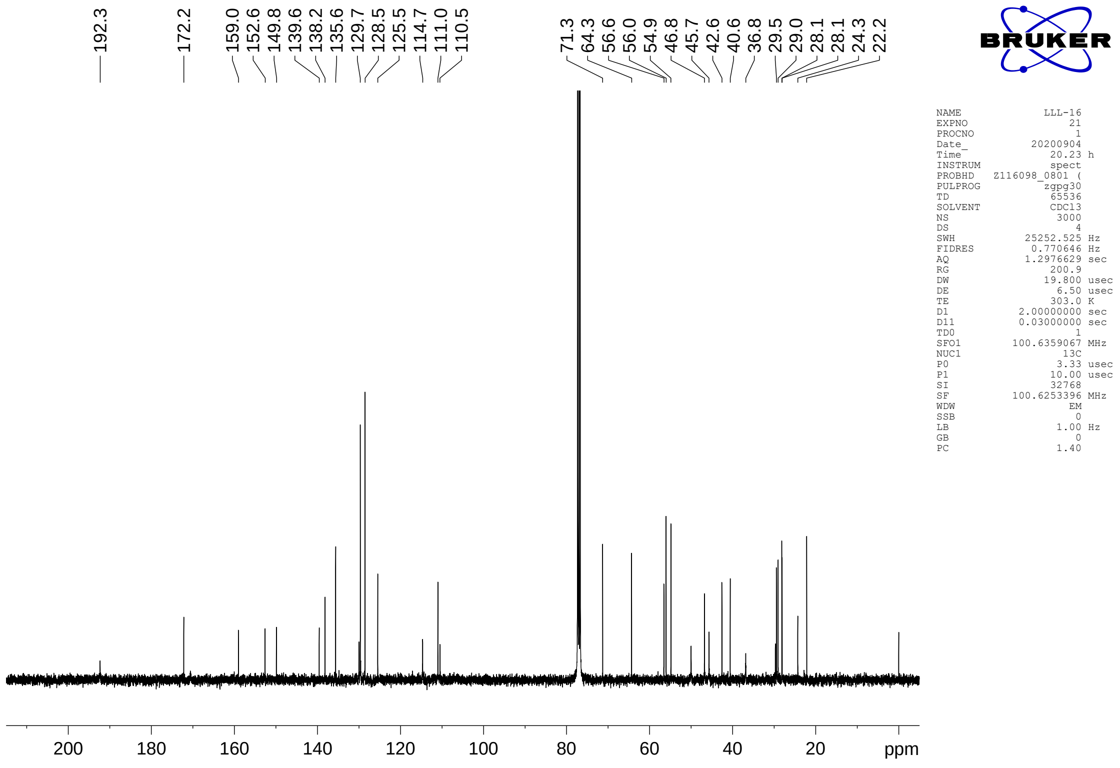

Figure S29. ^{13}C NMR of compound **7Da**

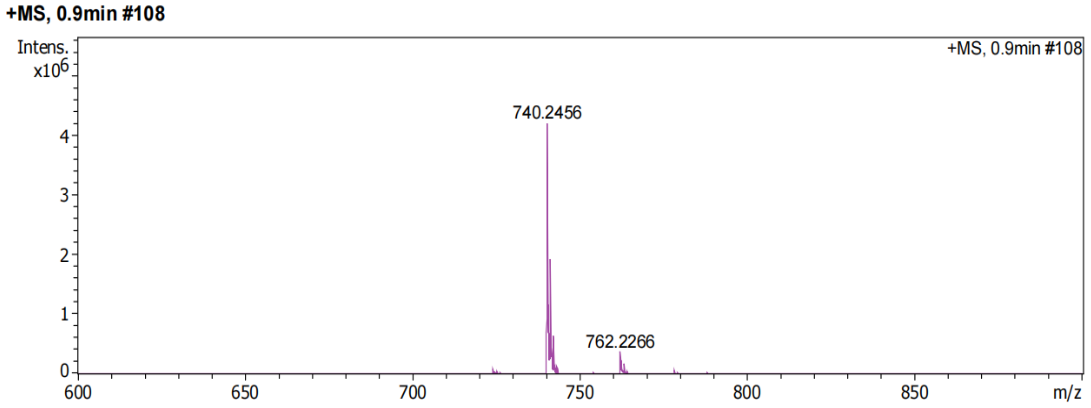

Figure S30. HRESIMS of compound **7Da**

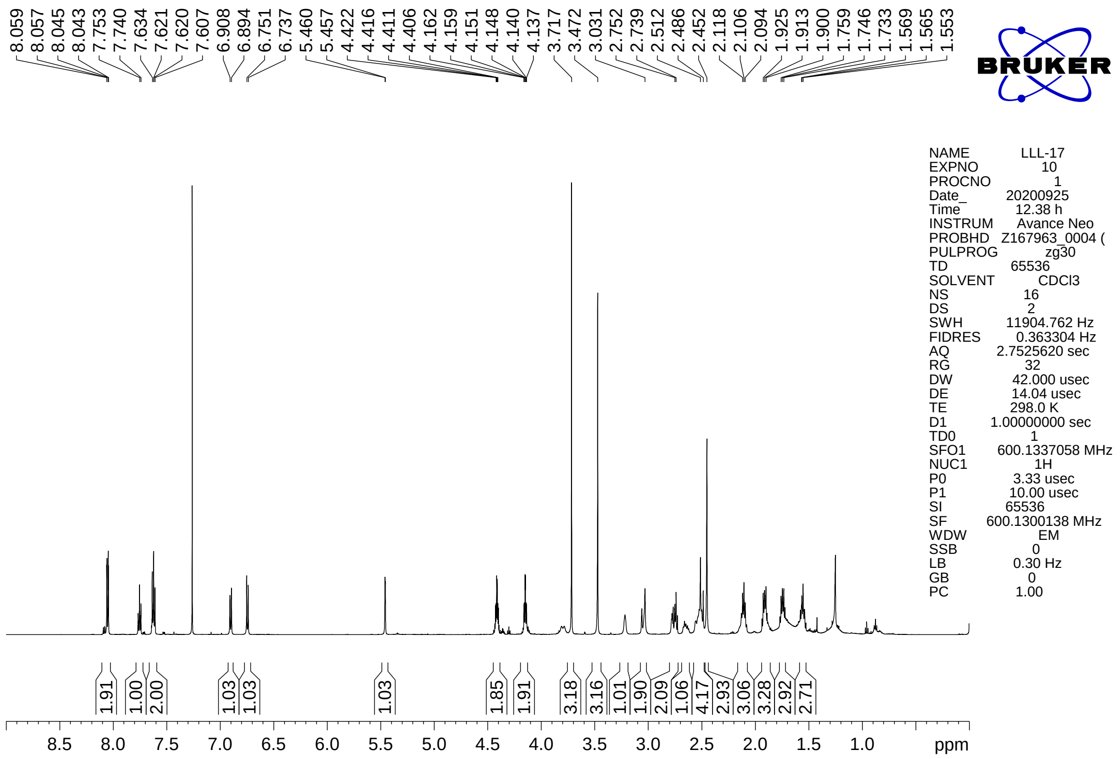

Figure S31. ^{1}H NMR of compound **7Db**

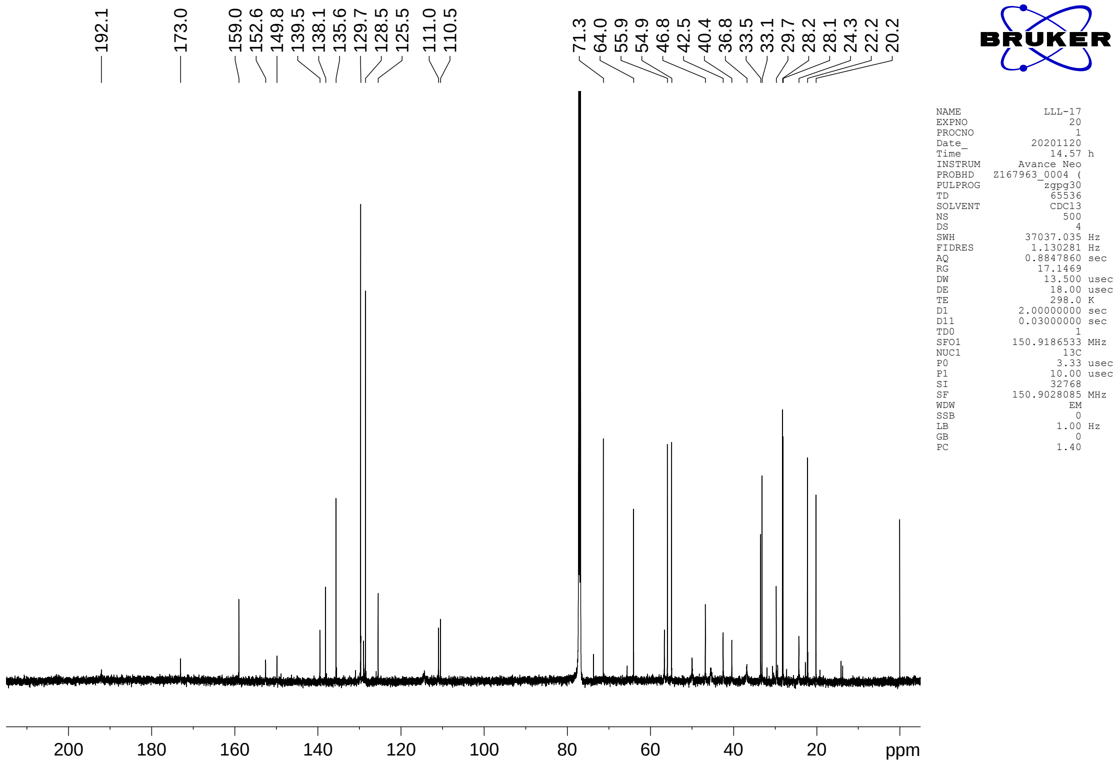

Figure S32. ^{13}C NMR of compound **7Db**

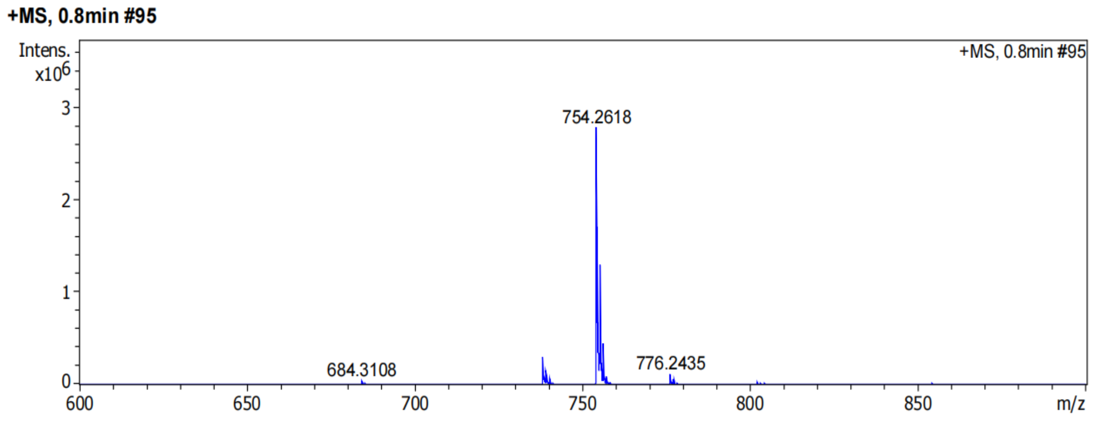

Figure S33. HRESIMS of compound **7Db**

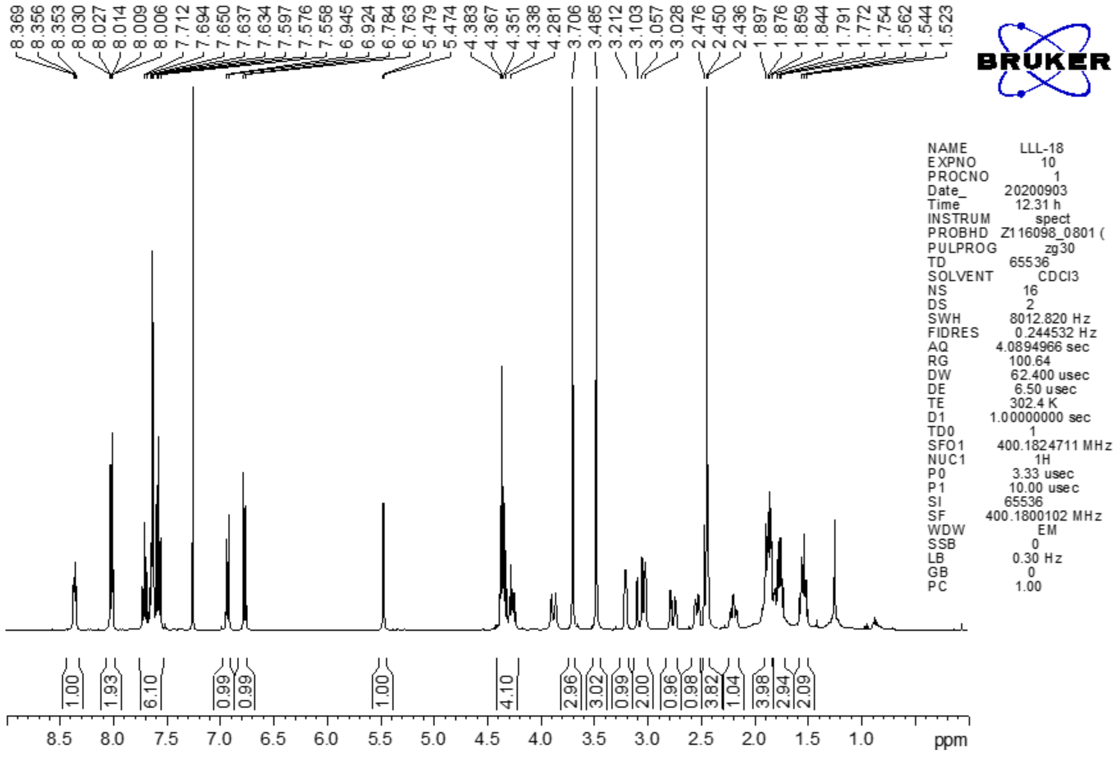

Figure S34. ^{1}H NMR of compound **7Dc**

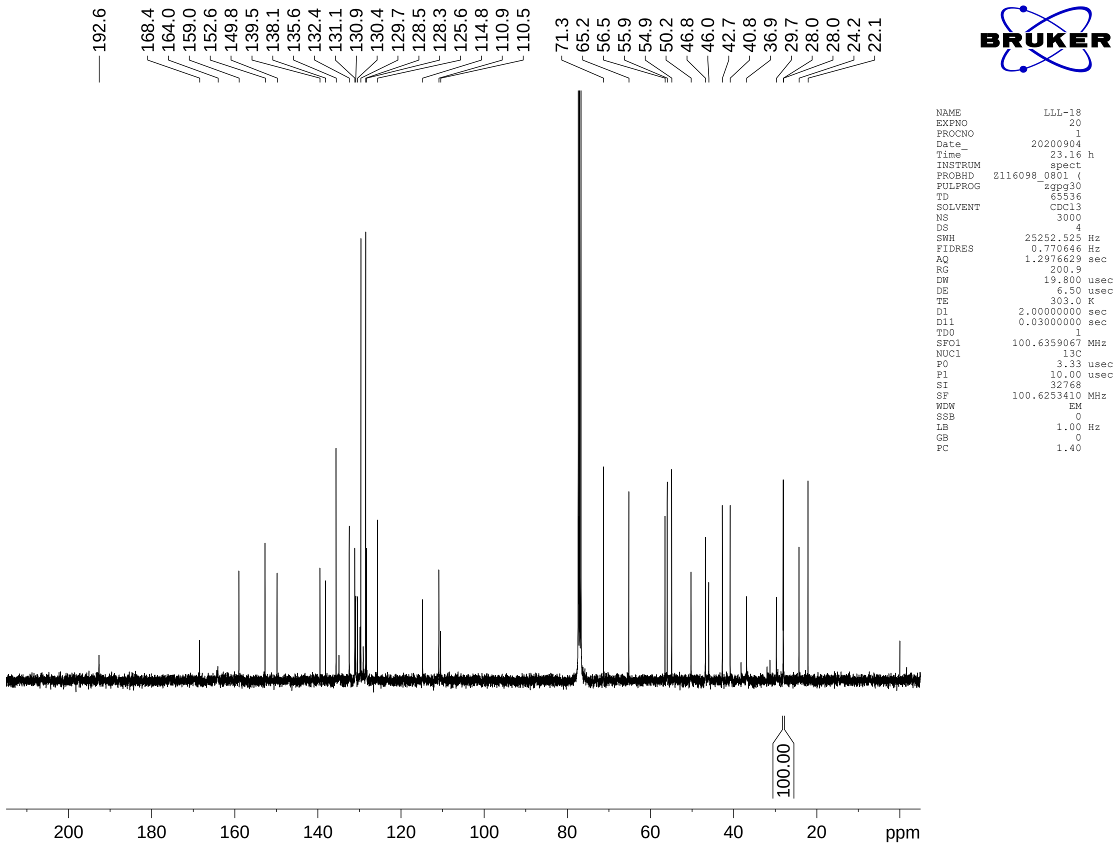

Figure S35. ^{13}C NMR of compound **7Dc**

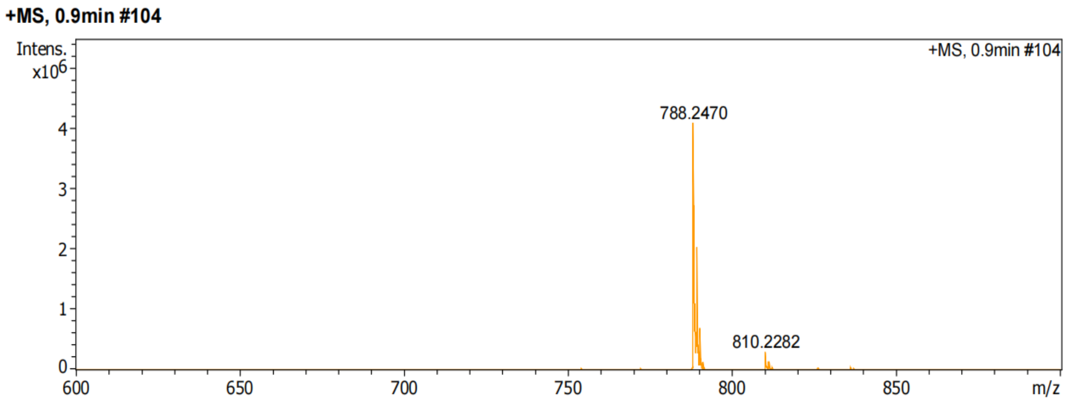

Figure S36. HRESIMS of compound **7Dc**

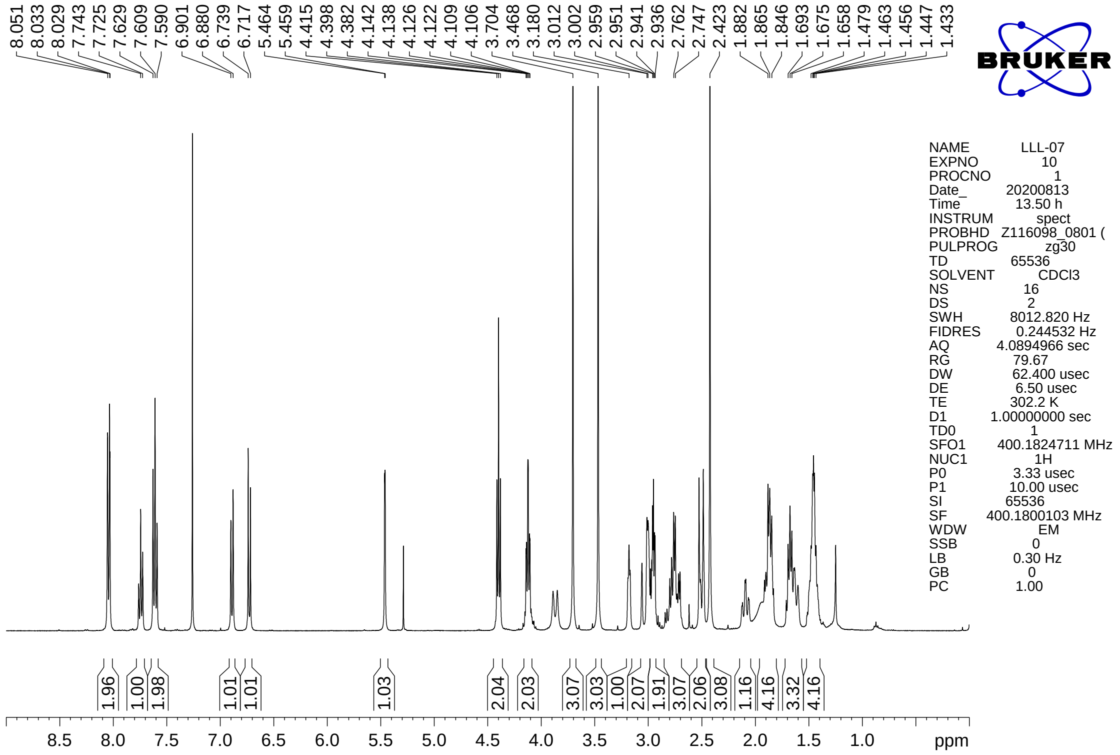

Figure S37. ^{1}H NMR of compound **7Ea**

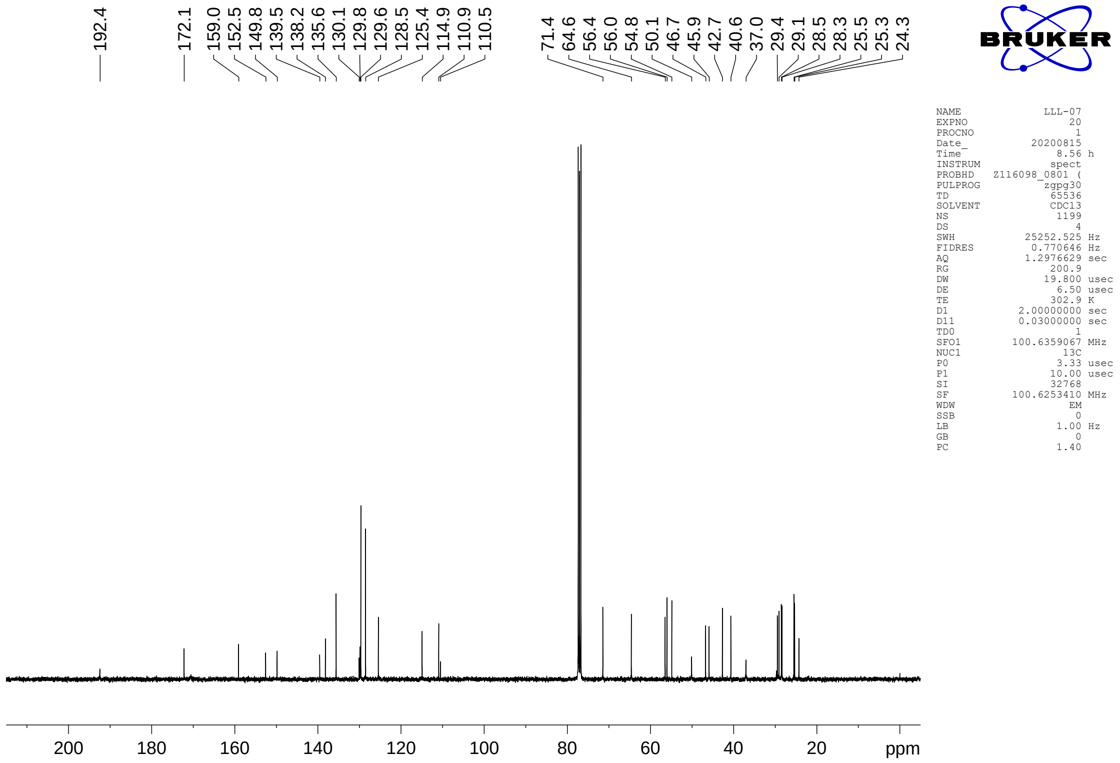

Figure S38. ^{13}C NMR of compound **7Ea**

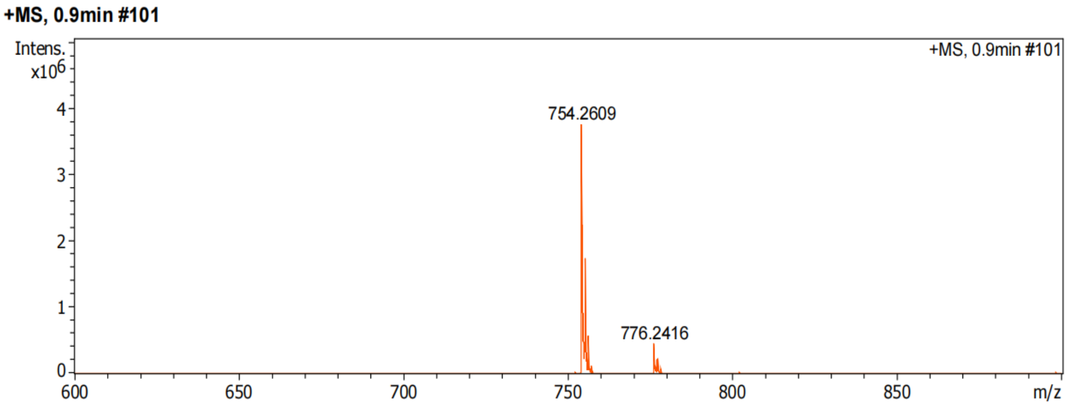

Figure S39. HRESIMS of compound **7Ea**

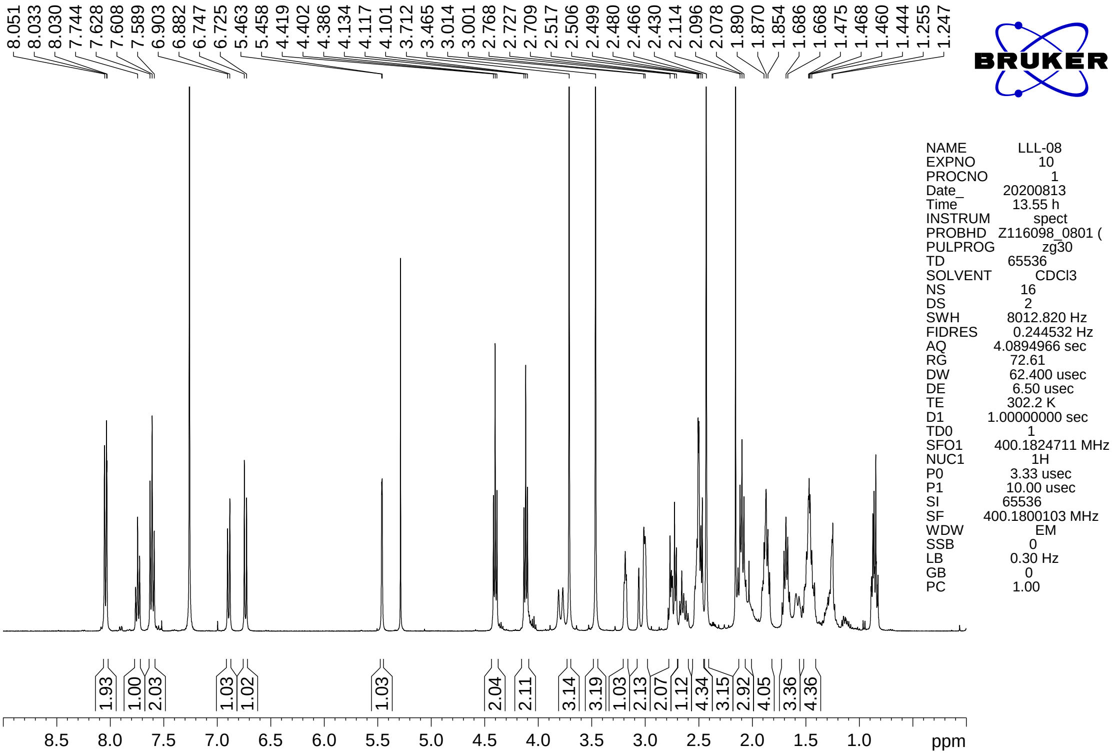

Figure S40. ^{1}H NMR of compound **7Eb**

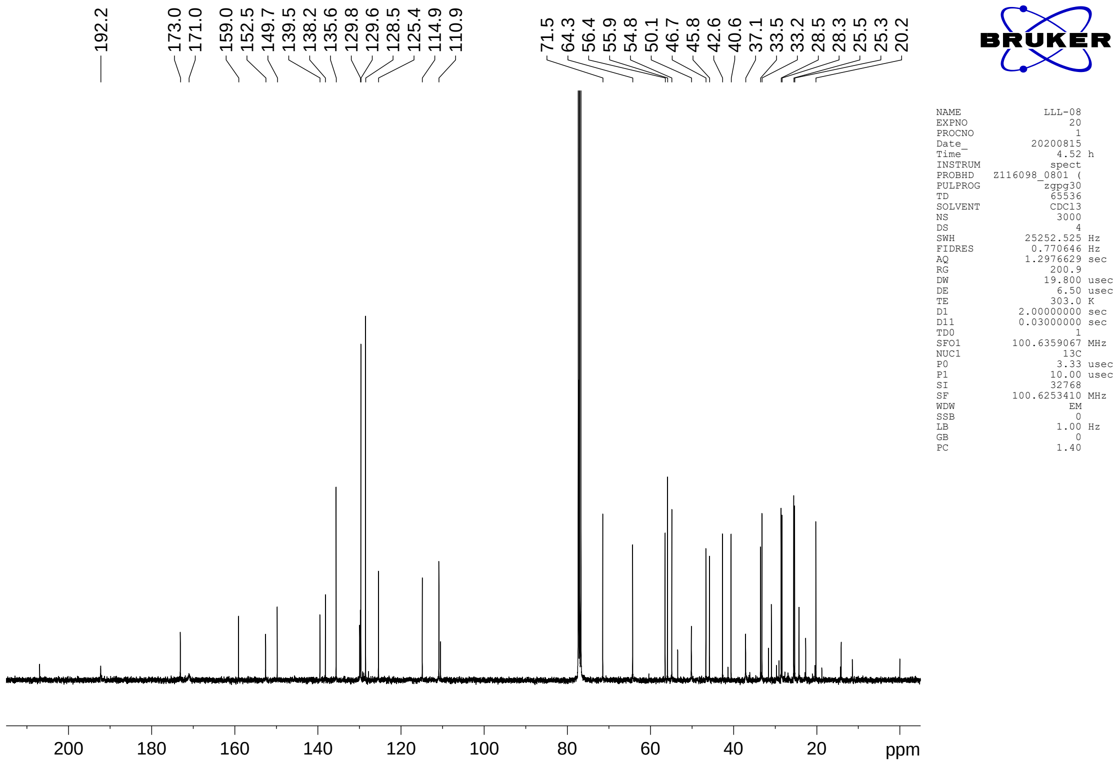

Figure S41. ^{13}C NMR of compound **7Eb**

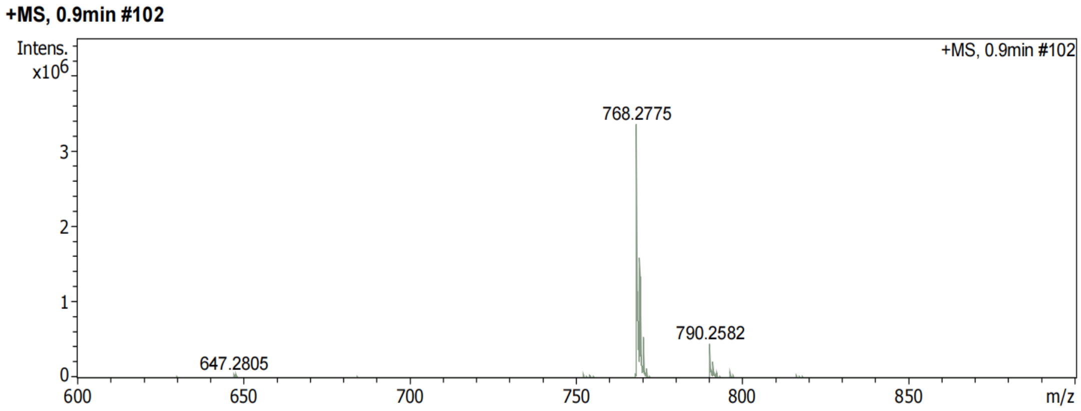

Figure S42. HRESIMS of compound **7Eb**

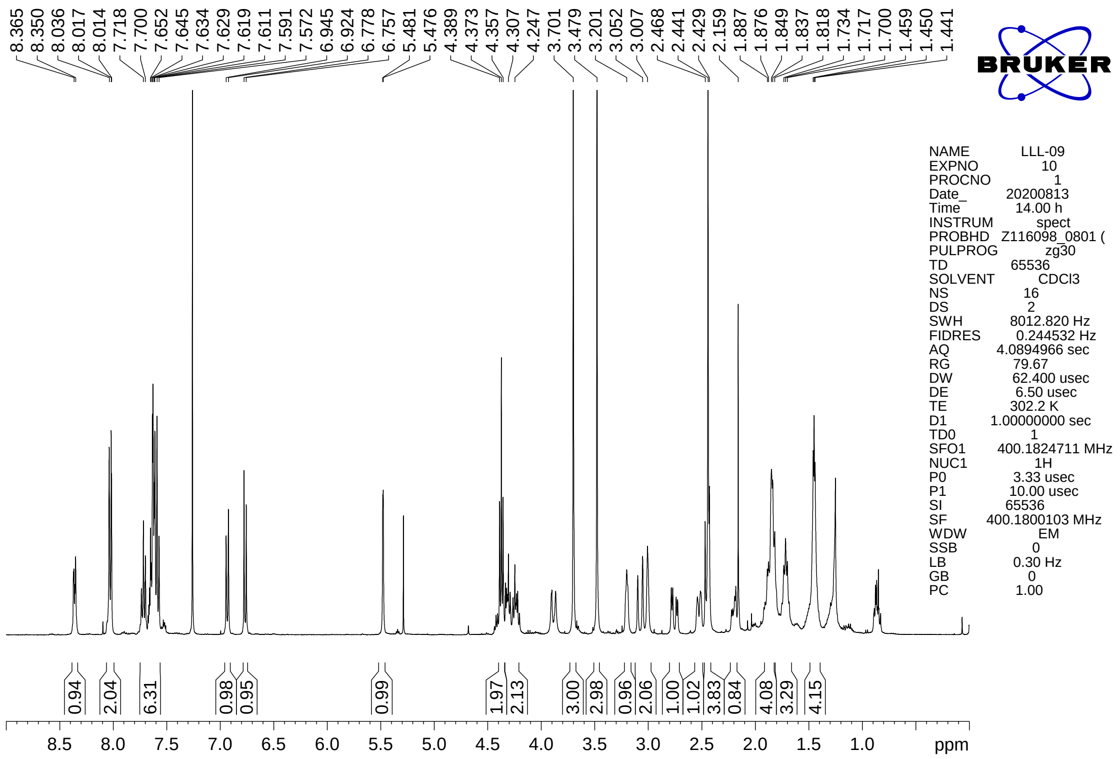

Figure S43. ^{1}H NMR of compound **7Ec**

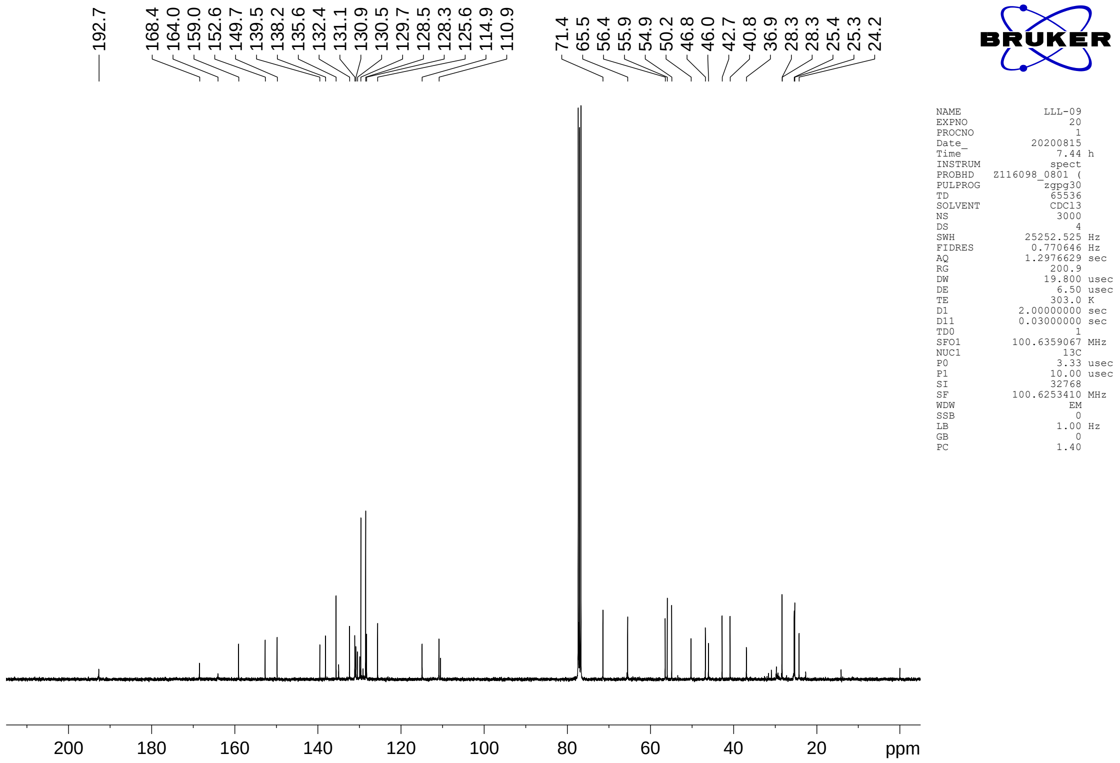

Figure S44. ^{13}C NMR of compound **7Ec**

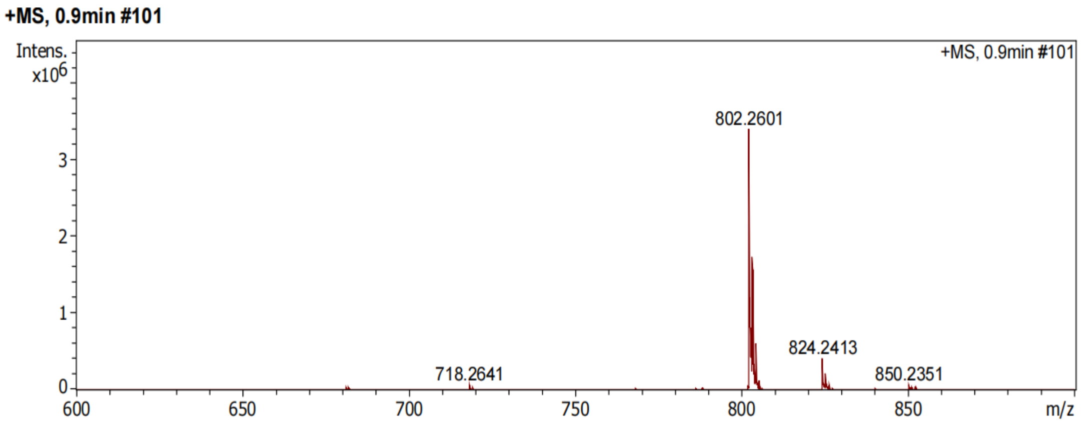

Figure S45. HRESIMS of compound **7Ec**

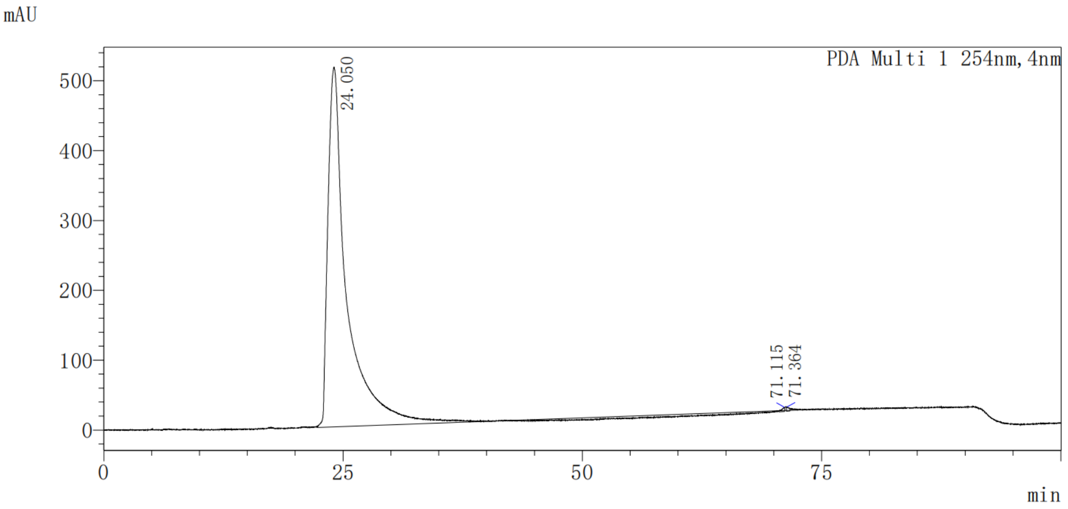

Results of analytical HPLC analysis of compound **7Cc** (254nm)

Peak	tR	S	H	S%
1	24.050	65369726	514335	99.639
2	71.115	125767	6083	0.192
3	71.364	111218	6065	0.170
Total		65606711	526483	

The analytical HPLC was a Shimadzu SPD-20A series equipped with an YMC C18 column (150 × 4.6 mm, 5 µm, 0.5 ml/min) using 85% aqueous MeOH as mobile phase to test compound **7Cc**.

Figure S46. Purity test of Compound **7Cc**
